# Anticancer effects of alpha-helical peptide epinecidin-1 and its variants in combination with doxorubicin

**DOI:** 10.1007/s12032-026-03344-0

**Published:** 2026-07-22

**Authors:** Sivakumar Jeyarajan, Sukumar Ranjith, Atchyasri Anbarasu, Indira Kandasamy, Prahalathan Chidambaram, Anbarasu Kumarasamy

**Affiliations:** 1 Microbial Biotechnology Laboratory, Department of Marine Biotechnology, Bharathidasan University, Tiruchirappalli, Tamil Nadu India; 2 Department of Molecular, Cellular and Developmental Biology, University of Michigan, Ann Arbor, MI USA; 3 Swamy Vivekananda Medical College Hospital and Research Institute, Tiruchengode, 637205 Tamil Nadu India; 4 Department of Biochemistry, Bharathidasan University, Tiruchirappalli, Tamil Nadu India

**Keywords:** Antimicrobial peptides, Epinecidin-1, Variants, Anticancer, Doxorubicin

## Abstract

In this study, we evaluated the in silico and in vitro anticancer activity of the antimicrobial peptide epinecidin-1 (Epi-1) and its lysine-substituted variants (Variant-1 (Var-1) & Variant-2 (Var-2)). Computational docking demonstrated energetically favourable and structurally consistent interactions between the peptides and cancer-associated receptors (MerTK (PDB ID: 7OLX), EphA3 (PDB ID: 2QO9), TGF-β receptor I/ ALK5 (PDB ID: 3TZM), TrkA / NTRK1 (PDB ID: 4AOJ), and progesterone receptor (PDB ID: 1A28)), with distinct binding orientations and interaction profiles observed across the variants. Molecular dynamics simulation further substantiated these findings by confirming the stability of the selected receptor-ligand complex, with consistent root mean square deviation (RMSD), root mean square fluctuation (RMSF), radius of gyration (Rg), solvent-accessible surface area (SASA), and intermolecular hydrogen-bond interactions profiles indicating sustained interaction integrity under dynamic conditions. The in vitro antiproliferative activity of Epi-1 and its variants was assessed by MTT assays against A549, HeLa, HepG2, IMR-32 and MCF-7 cell lines. Both variants exhibited a two- to four-fold increase in cytotoxic activity compared with native Epi-1. We also evaluated the combinational activity of each peptide with doxorubicin, where peptide-doxorubicin treatments resulted in effective cancer cell killing at reduced drug concentrations relative to individual treatments, the Var-2 + doxorubicin combination reduced cancer cell survival to below 10% at a combined concentration of 1 µg/mL (0.5 µg/mL peptide + 0.5 µg/mL doxorubicin), compared with ~ 30% survival in doxorubicin alone. This has been added alongside the existing two- to four-fold cytotoxicity enhancement of the variants over wild-type Epi-1, to better convey the impact of the synergistic response. While inducing selective lysis in cancer cells, the peptides exhibited minimal cytotoxicity toward non-cancerous HEK 293 cells, indicating improved therapeutic selectivity. DCFH-DA staining confirmed intracellular reactive oxygen species generation, and Acridine Orange/Ethidium Bromide (AO/EtBr) staining demonstrated apoptosis as the predominant mode of cell death across the cancer cell lines, although Var-1 induced necrotic death in HepG2 cells.

**Supplementary Information:**

The online version contains supplementary material available at https://doi.org/10.1007/s12032-026-03344-0.

## Introduction

In the current scenario, the chemotherapeutics have substantial toxic side effects along with their target function as anticancer drugs. In the drug resistance mechanisms, the chemotherapeutic drugs don’t specifically reach the cancer cells rather they accumulate in host normal cells or tracts, harming normal cells. The cancer cells develop resistance by adapting mechanisms like increased expression of drug-detoxifying enzymes, drug efflux channel proteins, alters the structure of receptor so the drug’s efficacy lose their potency to bind to its target receptors and also thrive the potential to cause defects in their cellular machinery, which leads to deregulation of cell death, ceases apoptosis which leads to increased survival of the cells [1]. Due to development of this skillful escaping mechanism the cancer cell has evaded to combat the action of drugs. Hence the treatment of cancer should be dealt with strategies which will overcome the resistance mechanism rather than to kill cancer cells. Therefore, it is an urge for scientists to elucidate the targets that are present on cancer cells and discern mechanism of binding to the targets.

Antimicrobial peptides (AMPs) exhibit potential antimicrobial and antitumor activities which have opened a new perspective for pharmaceutical applications [2, 3]. AMPs are also identified as a group of anticancer peptides that lack toxicity to normal mammalian cells and have superior mode of action that avoid the development of resistance in targeted cancer cells [4, 5] which makes them as an ideal therapeutic molecules. Hence AMPs are widely regarded as promising therapeutic agents against multidrug-resistant cancer cells. Nevertheless, only a few of them have entered into clinical trials and been commercialized, despite a comprehensive database cataloging large numbers of peptides identified and characterized from natural sources (http://aps.unmc.edu/AP/about.php*)* [6, 7]. This underlines the lack of exact information regarding the peptides stability, their mode of action and interaction with receptor molecules of different pathogens, tumor cells, etc. In addition to solid-phase synthesis, recombinant fusion strategies were also followed by many researchers. Formic acid cleavage in recombinant fusion peptides provides a practical route to biosynthesize short AMPs for research and translational development [8, 9].

Many variants of AMPs have been designed by amino acid substitution studies and validated by Bio-informatics approaches, Ex.- SVS-1, S1, dermaseptin B2, temporin-1CEa, magainin II, pleurocidin [4, 5, 10]. Our earlier computational docking studies have demonstrated that epinecidin-1 (Epi-1) and its variants (Var-1 & Var-2) exhibit significant binding affinity with the HER-2 receptor overexpressed in breast cancer cells, suggesting potential receptor-mediated uptake mechanisms in addition to membrane disruption [11]. It has also been studied that the majority of AMPs binds to the components of the cell membrane such as phosphatidylserine (PS), sialic acid or heparan sulfate which have differential expression between cancer and non-cancerous cells. So the cationic and amphipathic AMPs are able to discriminate between neoplastic and non-neoplastic cells [12], and interact specifically with the aforesaid negatively charged membrane components to kill cancer cells. Researchers have also investigated the receptor binding targets of AMPs to cancer cells. Ex. LfcinB binds to fibroblast growth factor (bFGF) and vascular endothelial growth factor receptor (VEGF) to inhibit growth of excess proliferating human endothelial cells in vitro [10]. NRC-03 and NRC-07 peptides (National Research Council peptides) (derived from pleurocidin) bind to P-glycoprotein (P-gp) present on breast carcinoma cells and prevents their growth in xenografted mice. P-gp is an ATP-dependent drug efflux pump responsible for discharging toxic substances from cancer cells. This way, the cancer cells flee from the effect of cancer drugs. The presence of AMPs inhibits the action of efflux pump so that the cancer drug stays within the cell for a long time and deploys its effect [13, 14].

Epi-1 is a promising AMP found in leukocytes of marine grouper fish and has been reported to exhibit antibacterial and antifungal activity [15, 16]. The engineered Epi-1 variants exhibit enhanced stability, antibacterial and antifungal activity when compared with their wildtype counterpart [17], In this study, Epi-1 and lysine-substituted variants were evaluated in different cancer cells alone and with doxorubicin to test whether co-treatment enhances cytotoxicity and apoptotic signaling across A549, HeLa, HepG2, IMR-32, and MCF-7, with HEK-293 selectivity profiling.

## Materials and methods

**Fig. 1:**
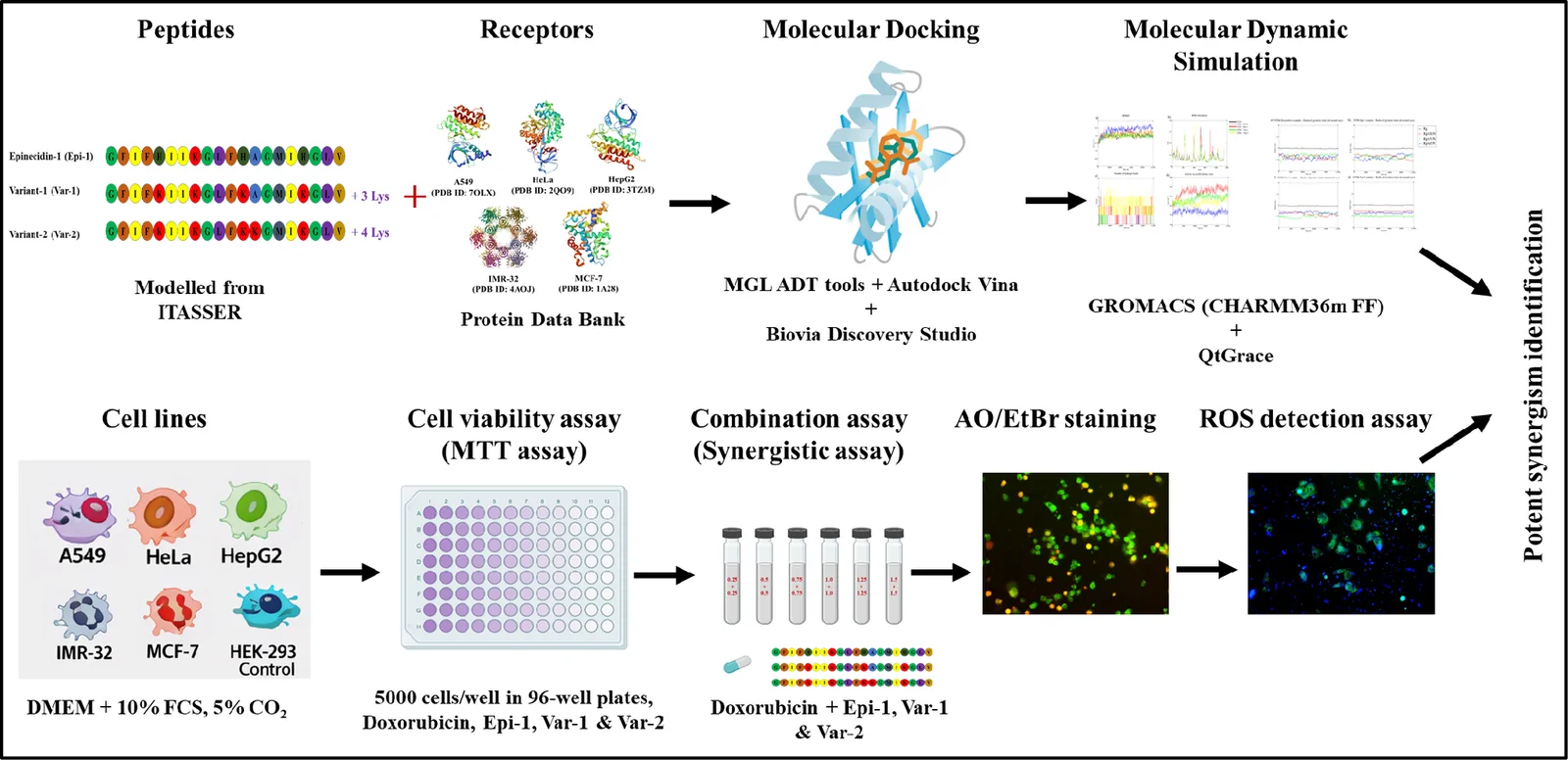
Schematic overview of integrated in silico and in vitro workflow for the synergistic anticancer evaluation of Epi-1 peptide and its variants

The overall experimental strategy adopted in this study, integrating computational docking and molecular dynamics simulations with subsequent *in vitro* validation, is illustrated in Fig. 1.

### Molecular docking

#### Peptides

Antimicrobial peptides (AMPs) Epi-1 and its variants (Var-1 & Var-2) (purity ≥ 95%) were chemically synthesized using 9-fluorenylmethoxycarbonyl (Fmoc) solid-phase peptide synthesizer by GenicBio Limited, Shanghai, China. The wildtype peptide Epi-1 (GFIFHIIKGLFHAGKMIHGLV) served as the structural framework for the rational design of lysine-enriched analogues aimed at augmenting net positive charge and amphipathicity. In Var-1, histidine residues at positions 5, 12, and 18 were substituted with lysine (H5K, H12K & H18K), thereby enhancing cationicity and maintaining the intrinsic α-helical conformation. Var-2 was derived from the Var-1 backbone through an additional alanine-to-lysine substitution at position 13 (A13K), generating a more positively charged analogue to evaluate the membrane-disruption activity–associated anticancer efficacy. All peptides were synthesized *via* standard Fmoc solid-phase peptide synthesis with ≥ 95% purity, and the complete sequences with designated substitution sites are summarized in Table 1, consistent with previously established methodologies [18].

**Table 1:** Sequence of Epi-1 and its variants. Lysine replaced residues are highlighted, bold, italicized, and underlined

Peptide name	Sequence
Epinecidin-1	GFIFHIIKGLFHAGKMIHGLV
Variant-1- Replacement of H with K	*GFIF***K***IIKGLF***K***AGKMI***K***GLV
Variant-2- Replacement of A with K	*GFIFKIIKGLFK***K***GKMIKGLV

*Replaced residues are represented in bold letters and underlined.

#### Protein and ligand preparation

The peptides Epi-1 and its lysine-substituted variants (Var-1 & Var-2) were taken as ligands for molecular docking. Their modelled PDB structures, generated by the I-TASSER platform, were energy-minimized prior to docking [19, 20]. The peptide structures used for docking were derived from our previously published and validated I-TASSER models; de novo structural modeling was not performed in the present study.

The reference chemotherapeutic compound doxorubicin in PDB format was taken from the DrugBank database (https://go.drugbank.com/). All peptide ligands were prepared using MGL AutoDock Tools (ADT) v1.5.7, which involved the addition of polar hydrogens, assignment of charges and conversion to PDBQT format [21].

#### Receptor preparation

The receptor proteins corresponding to major oncogenic targets in each cancer cell line were retrieved from the Protein Data Bank (https://www.rcsb.org/*)* Table 2. For each receptor, water molecules, co-crystallized ligands, and heteroatoms were removed, polar hydrogens were added and Kollman charges were assigned. All receptor structures were saved in PDBQT format.

#### Grid generation and docking protocol

Docking simulations were performed using AutoDock Vina v1.2.0 executed through the standalone Command-Prompt interface [22]. For each receptor, a grid box was centered on the co-crystallized ligand pocket or catalytic region, and grid dimensions (Å) were optimized to encompass the entire active site and permit full ligand flexibility. Each configuration file contained receptor and ligand names, grid coordinates, and output directories for reproducibility. Docking runs were automated using batch scripts.

The docking output was ranked based on binding free energy (ΔG, kcal/mol), and the best-scoring pose for each complex was retained for interaction analysis.

**Table 2:** Curated receptor targets with PDB identifiers for docking in the cancer cell models used in this study

Cell line	Receptor target	Justification for inclusion in anticancer docking	Reference
A549 (non-small cell lung carcinoma)	MerTK kinase domain (PDB ID: 7OLX)	MerTK, part of the TAM receptor family, promotes cell survival, epithelial–mesenchymal transition, and immune suppression. Docking peptides to MerTK aids in identifying molecules that could block pro-survival or pro-metastatic signaling in NSCLC.	[23]
HeLa (cervical carcinoma)	EphA3 receptor kinase domain (PDB ID: 2QO9)	Eph receptors are overexpressed in epithelial tumors, modulating angiogenesis, adhesion, and migration. Targeting EphA3 kinase domains provides insights into anti-migratory and anti-invasive potential.	[24]
HepG2 (hepatocellular carcinoma)	TGF-β receptor I/ALK5 kinase domain (PDB ID: 3TZM)	The TGF-β/SMAD pathway drives tumor progression and metastasis in advanced liver cancer. Inhibiting ALK5 can suppress epithelial-to-mesenchymal transition and fibrosis-associated tumor growth.	[25]
IMR-32 (neuroblastoma)	TrkA/NTRK1 kinase domain (PDB ID: 4AOJ)	TrkA signaling regulates neuronal differentiation and survival; aberrant activation contributes to neuroblastoma aggressiveness. Peptide binding at the TrkA ATP site can potentially disrupt oncogenic signaling cascades.	[26]
MCF-7 (breast carcinoma)	Progesterone receptor (PDB ID: 1A28)	PR signaling cross-talks with ERα pathways to regulate proliferation, apoptosis, and therapeutic response. Docking against the PR LBD allows assessment of peptide interference in hormone-driven oncogenic signaling.	[27]

#### Interaction analysis and visualization

All docked complexes were analyzed using BIOVIA Discovery Studio Visualizer v24.1.0.23298 to identify hydrogen bonding, hydrophobic interactions, electrostatic complementarity, and key active-site residues. The visualized complexes were rendered for both two- and three-dimensional representations.

### Molecular dynamics simulation

To evaluate the structural stability of receptor-ligand complexes predicted by molecular docking, molecular dynamics (MD) simulations were performed using the GROMACS 2024.4 simulation package on a Ubuntu 24.04 LTS Linux platform with GPU acceleration enabled environment [28]. The protein and peptide components were parameterized using the CHARMM36m force field [29], while ligand parameters for doxorubicin was generated using the CHARMM General Force Field (CGenFF) [30].

Docking analyses were initially performed against five oncogenic receptor systems relevant to different cancer cell types, including MerTK (PDB ID: 7OLX), EphA3 (PDB ID: 2QO9), TGF-β receptor I/ALK5 (PDB ID: 3TZM), TrkA/NTRK1 (PDB ID: 4AOJ), and the progesterone receptor (PDB ID: 1A28). Among these targets, the TGF-β receptor I kinase domain (TGF-β receptor I/ALK5; PDB ID: 3TZM) displayed the most favourable docking energies and consistent interaction patterns with the peptide ligands, particularly with Var-2 (ΔG ≈ − 9.5 kcal/mol) and doxorubicin (ΔG ≈ − 11.1 kcal/mol). Given the central role of TGF-β receptor I/ALK5 in TGF-β-mediated oncogenic signaling pathways associated with tumor proliferation and metastasis, this receptor was selected as the representative system for detailed MD evaluation.

The docked complexes consisting of TGF-β receptor I/ALK5 with doxorubicin, Epi-1, Var-1, and Var-2 were subjected to atomistic MD simulations to assess the stability of the predicted binding poses. Each complex was placed in a cubic simulation box with a minimum distance of 10 Å between the protein surface and box boundary. The systems were solvated using TIP3P water model, and counter ions were introduced to neutralize the net charge.

Energy minimization was performed using the steepest-descent algorithm until maximum force on the system fell below 1000 kJ mol⁻¹ nm⁻¹. Subsequently, the systems were equilibrated in two sequential phases. Temperature stabilization was first carried out under constant volume conditions (NVT ensemble) for 100 ps at 300 K using the V-rescale thermostat. Pressure equilibration followed under constant pressure conditions (NPT ensemble) for 100 ps at 1 bar using the Parrinello-Rahman barostat.

Following equilibration, production MD simulations were performed for 100 ns with an integration time step of 2 fs. Periodic boundary conditions were applied in all spatial directions, and long-range electrostatic interactions were calculated using the Particle Mesh Ewald method [31].

Trajectory analyses were conducted using the integrated analysis tools of GROMACS to evaluate key structural descriptors, including root mean square deviation (RMSD), root mean square fluctuation (RMSF), radius of gyration (Rg), solvent-accessible surface area (SASA), and intermolecular hydrogen-bond interactions. These parameters were used to assess conformational stability and ligand retention within the receptor binding pocket throughout the simulation trajectory. Graphical representations of the simulation trajectories were generated using QtGrace (Grace-5.1.22/QtGrace v0.2.6).

### Cell lines

A549 (lung cancer), HeLa (cervical cancer), HepG2 (liver cancer), IMR-32 (human neuroblastoma), MCF-7 (breast cancer) and HEK 293 (Human Embryonic Kidney) cells were cultured in Dulbecco’s modified Eagle’s medium (DMEM) supplemented with 10% fetal calf serum (FCS), antibiotics penicillin (5 mg/mL) and streptomycin (1 mg/mL). The cells were obtained from National Centre for Cell Science, Pune, India.

### Cell viability assay (MTT)

Cells were cultured at 5000 cells/well in 96-well plates and treated with different concentrations of Epi-1, Var-1 and Var-2, and doxorubicin for 24 h with concentration ranging from (0.5, 1, 1.5, 2, 2.5 and 3 µg/ml). The number of viable cells in each well was estimated by adding 100 µl of 2.5 mg of 3-(4,5-dimethylthiazol-2-yl)−2,5-diphenyl tetrazolium bromide (MTT) (Sigma, Louis, MO) in 2.5mL PBS + 7.5 mL DMEM and incubated for 3 h at 37 °C [32]. After 3 h the MTT mixture was removed, and resulting formazan crystals were dissolved in 100 µl of Methanol and Dimethyl sulfoxide (50:50) [33]. The optical density was measured spectrophotometrically at 570 nm on a microtiter plate reader (iMark Bio-Rad USA). Experiments were done in triplicates. Results are expressed as percentage of survival cells with values of DMEM treated control cells. For non-cancerous cells, HEK was used to determine the drug molecule effect on normal cells in vitro. The experiments were performed in triplicates. Tukey’s multiple comparisons test was performed for statistical analysis and significance representations are a; *p* < 0.5, b; *p* < 0.1, c; *p* < 0.01, and d; *p* < 0.001. The non-significance is not shown.

### Combination assay of Epi-1, Var-1 and Var-2 with doxorubicin

For synergistic assay, Epi-1 + doxorubicin, Var 1 + doxorubicin and Var 2 + doxorubicin were added in equal amount to a concentration of 0.5, 1, 1.5, 2, 2.5 and 3 µg/mL [34]. Example 0.5 µg/mL contains 0.25 µg/mL peptide + 0.25 µg/mL doxorubicin. MTT assay was done for HeLa, IMR-32, MCF-7, HepG2 and A549 cells in 96-well culture dishes after treatment with combinations. Cells with DMEM alone without any peptide served as control [35].

The combination interaction was quantified by Bliss independence using Graphpad prism.

YA = 1/(1 + 10^((LogEC50A - X) * HillSlope)).

YB = 1/(1 + 10^((LogEC50B - X) * HillSlope)).

< A > Y = YA.

< B > Y = YB.

< C > Y = YA + YB - YA * YB.

YA is fractional response to drug A alone (peptides in this study). YB is fractional response to drug B alone (doxorubicin).

The obtained LogEC 50 values for the concentrations used in this study is shown in Table 4.

### Detection of cell variations by acridine orange (AO)/ethidium bromide (EtBr) AO/EtBr staining

HeLa, IMR-32, HepG2 and A549 cells were grown to 90% confluence (5000 cells/well) in 96-well plates. For AO/EtBr staining, cultures were treated with 3 µg/mL of Epi-1, Var-1, Var-2 and doxorubicin for 24 h and processed for staining using PBS containing 1 µg/mL EtBr and 1 µg/mL AO [36, 37]. Cells were washed with PBS alone to remove the excess dye, subsequently observed and pictured under a FLoid Cell Imaging Station (Life Technologies) fluorescence microscope [38].

### Detection of reactive oxygen species (ROS) detection in HeLa cells

The formation of endogenous ROS in HeLa cells after treatment with the peptides were evaluated by staining with 2’, 7’ – dichlorodihydrofluorescein diacetate (DCFH-DA) as described earlier [39, 40] and viewing under fluorescence microscope [41]. Briefly, after treatment with the peptides at 3 µg/mL for 6 h, the cells were washed with PBS and incubated with 10 µM DCFH-DA for 30 min at 37 °C in the dark condition. DCFH-DA has the ability to diffuse into the cell and is deacetylated by cellular esterases to a non-fluorescent compound, 2’,7’ - dichlorodihydrofluorescein (DCFH). DCFH is rapidly oxidized by ROS to the highly fluorescent 2’, 7’ - dichlorofluorescein (DCF). The fluorescence intensity is proportional to the ROS levels within the cell cytosol and can be easily seen under a fluorescent microscope with excitation and emission wavelengths at 485 and 535 nm, respectively [42]. Doxorubicin at 3 µg/mL and 5mM H_2_O_2_ were treated separately as control for the induction of ROS.

## Results

### Molecular docking results

**Fig. 2:**
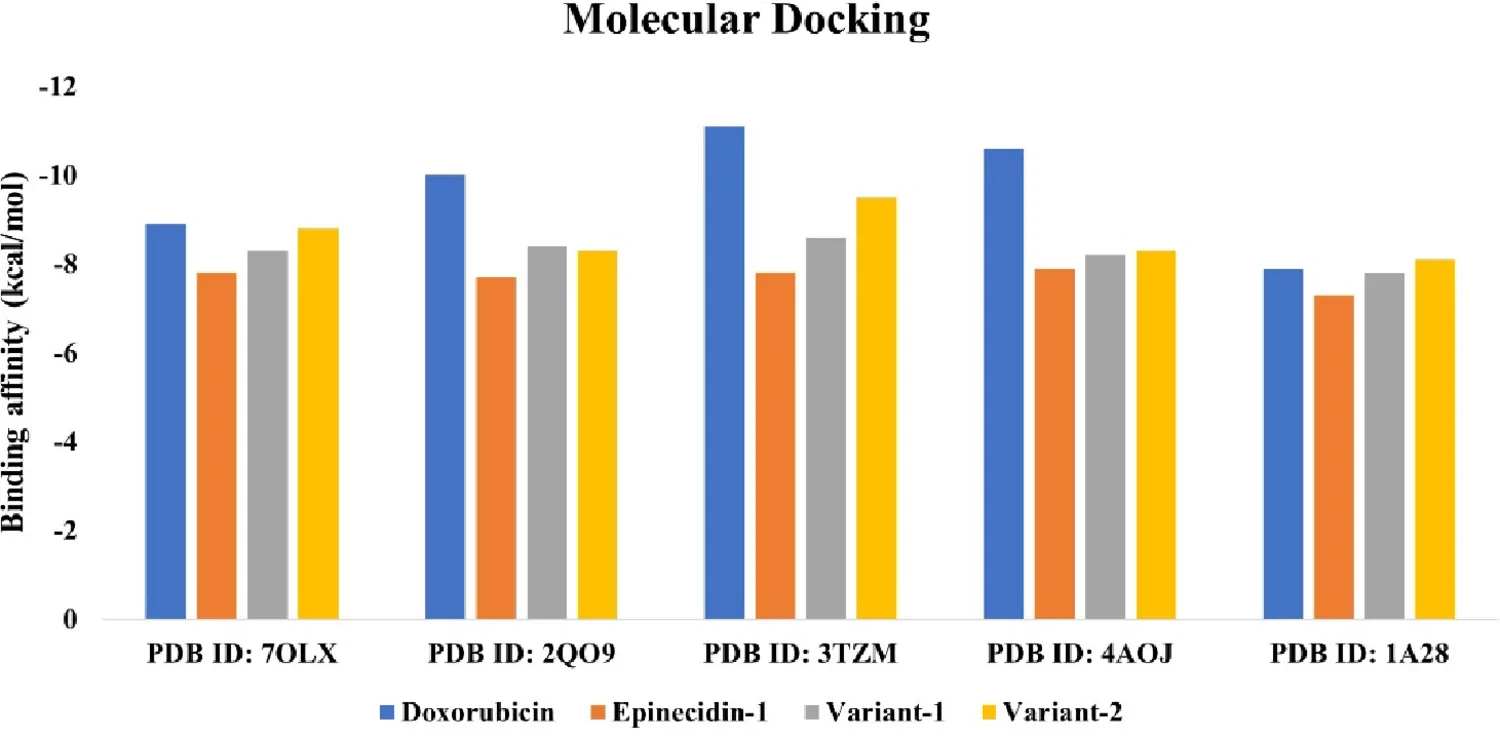
Docking scores (free energy change ΔG, kcal/mol) of doxorubicin, Epi-1, and its variants against receptor targets

Molecular docking analysis was performed to predict the binding interactions of Epi-1, and its lysine-substituted variants (Var-1 & Var-2), and the reference drug doxorubicin with key oncogenic receptors representing each cancer model-MerTK (7OLX, A549), EphA3 (2QO9, HeLa), TGF-β receptor I/ALK5 (3TZM, HepG2), TrkA/NTRK1 (4AOJ, IMR-32), and the progesterone receptor ligand-binding domain (1A28, MCF-7). All ligands exhibited thermodynamically favorable binding with predicted free energies (ΔG) ranging from − 7.3 to − 11.1 kcal/mol, confirming the formation of stable receptor–ligand complexes (Fig. 2).

Among the peptide ligands, Var-2 consistently demonstrated higher binding affinity than Epi-1 and Var-1, while doxorubicin maintained the most favorable ΔG values across the receptors (Table 3). In the MerTK receptor of A549, Var-2 exhibited strong binding (ΔG ≈ − 8.8 kcal/mol), followed by Var-1 (–8.3 kcal/mol) and Epi-1 (–7.8 kcal/mol). A similar affinity pattern was observed in the EphA3 receptor of HeLa cells, where Var-2 (–8.3 kcal/mol) and Var-1 (–8.4 kcal/mol) formed stable complexes, while Epi-1 displayed lower binding strength (–7.7 kcal/mol).

Docking with the TGF-β receptor I/ALK5 of HepG2 yielded the most stable complexes, with doxorubicin showing the lowest free energy (–11.1 kcal/mol). Var-2 (–9.5 kcal/mol) exhibited favorable hydrogen-bonding and electrostatic interactions, while Var-1 (–8.6 kcal/mol) and Epi-1 (–7.8 kcal/mol) showed moderate stabilities. For the TrkA/NTRK1 receptor of IMR-32, doxorubicin again displayed high affinity (–10.6 kcal/mol), whereas Var-2 (–8.3 kcal/mol) and Epi-1 (–7.9 kcal/mol) exhibited stable but less favorable binding energies.

In the progesterone receptor of MCF-7, Var-2 demonstrated the strongest peptide–receptor interaction (–8.1 kcal/mol), followed by Var-1 (–7.8 kcal/mol) and Epi-1 (–7.3 kcal/mol); doxorubicin bound at − 7.9 kcal/mol through hydrophobic stabilization within the ligand cavity. These results collectively indicate that lysine substitution enhances electrostatic complementarity and hydrogen-bond formation, improving receptor binding affinity without altering α-helical stability. Detailed residue-level interactions and corresponding 2D docking images are provided in the Supplementary data file.

**Table 3:** Predicted binding free energies (ΔG, kcal/mol) of doxorubicin, Epi-1, and its variants docked against receptor targets of A549, HeLa, HepG2, IMR-32, and MCF-7 cell lines

PDB ID	7OLX	2QO9	3TZM	4AOJ	1A28
Receptor	MerTK kinase domain	EphA3 receptor kinase domain	TGF-β receptor I/ALK5 kinase domain	TrkA/NTRK1 kinase domain	Progesterone receptor
Representative cancer cell line	A549	HeLa	HepG2	IMR-32	MCF-7
Doxorubicin	−8.9	−10	−11.1	−10.6	−7.9
Epinecidin-1	−7.8	−7.7	−7.8	−7.9	−7.3
Variant-1	−8.3	−8.4	−8.6	−8.2	−7.8
Variant-2	−8.8	−8.3	−9.5	−8.3	−8.1

### Molecular dynamics simulation analysis

To further evaluate stability of the docked complexes, molecular dynamics simulations were performed for the receptor selected from docking analysis. Among the five receptors initially screened MerTK (7OLX), EphA3 (2QO9), TrkA/NTRK1 (4AOJ), the progesterone receptor (1A28), and the TGF-β receptor I kinase domain (3TZM), the 3TZM system demonstrated the most favourable binding affinities and consistent interaction patterns with both the reference drug and peptides. Consequently, the 3TZM receptor was selected for detailed simulation analysis.

**Fig. 3:**
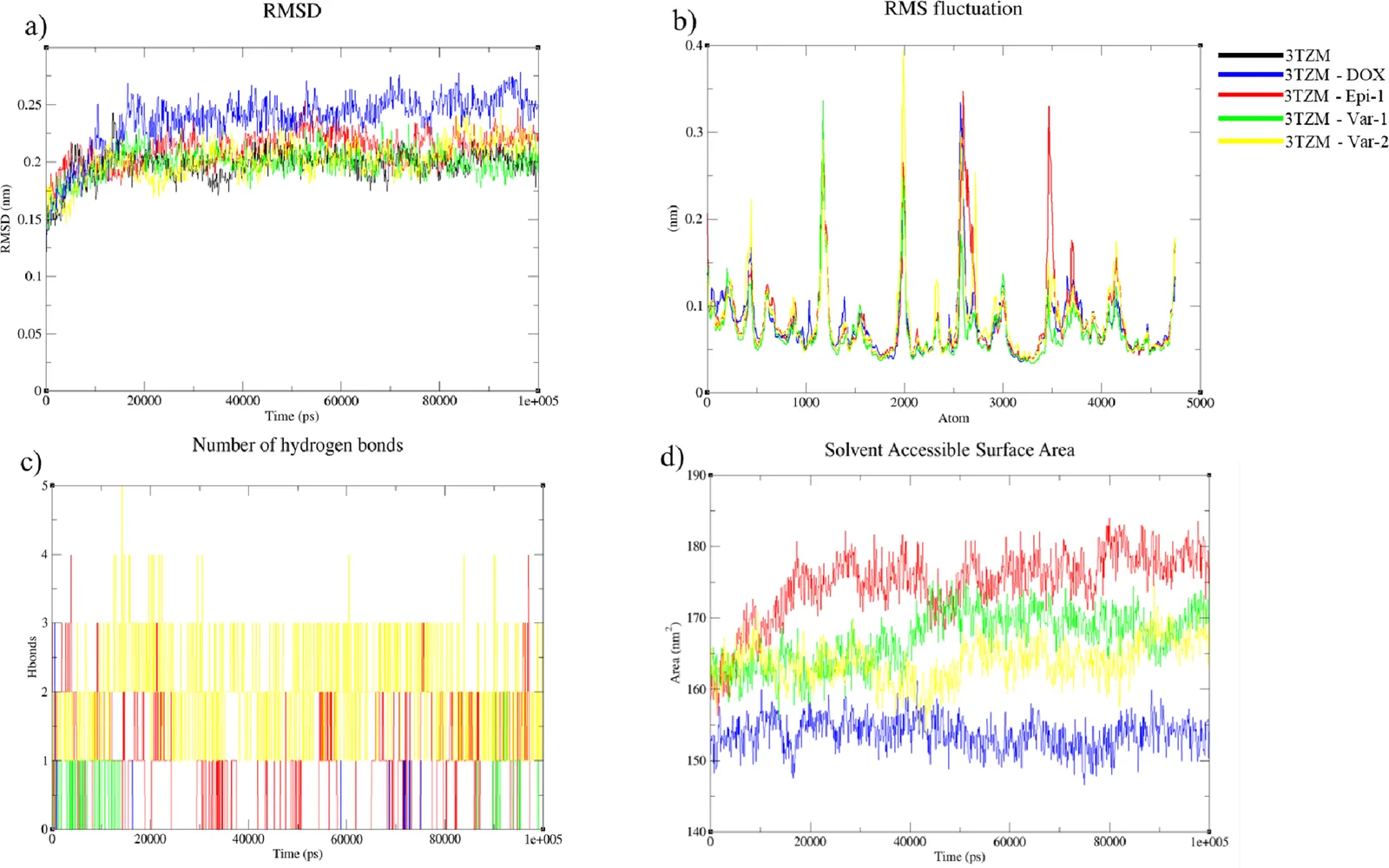
Molecular dynamics simulation analysis of 3TZM–ligand complexes over 100 ns. (**a**) Backbone root mean square deviation (RMSD) profiles of the 3TZM receptor in complex with doxorubicin, Epi-1, Var-1, and Var-2 demonstrate rapid equilibration followed by stable trajectories, indicating maintenance of overall structural integrity. (**b**) Root mean square fluctuation (RMSF) analysis reveals that residue mobility is predominantly confined to loop and terminal regions, while residues within the catalytic pocket remain comparatively rigid, suggesting preservation of functional stability upon ligand binding. (c) Time evolution of intermolecular hydrogen bonds indicates sustained receptor-ligand interactions across all systems, with variations in bond occupancy reflecting differences in interaction persistence among the peptide variants. (d) Solvent accessible surface area (SASA) profiles exhibit minimal fluctuations throughout the simulation, implying that ligand association does not induce significant conformational rearrangements or alter the global surface exposure of the receptor

**Fig. 4:**
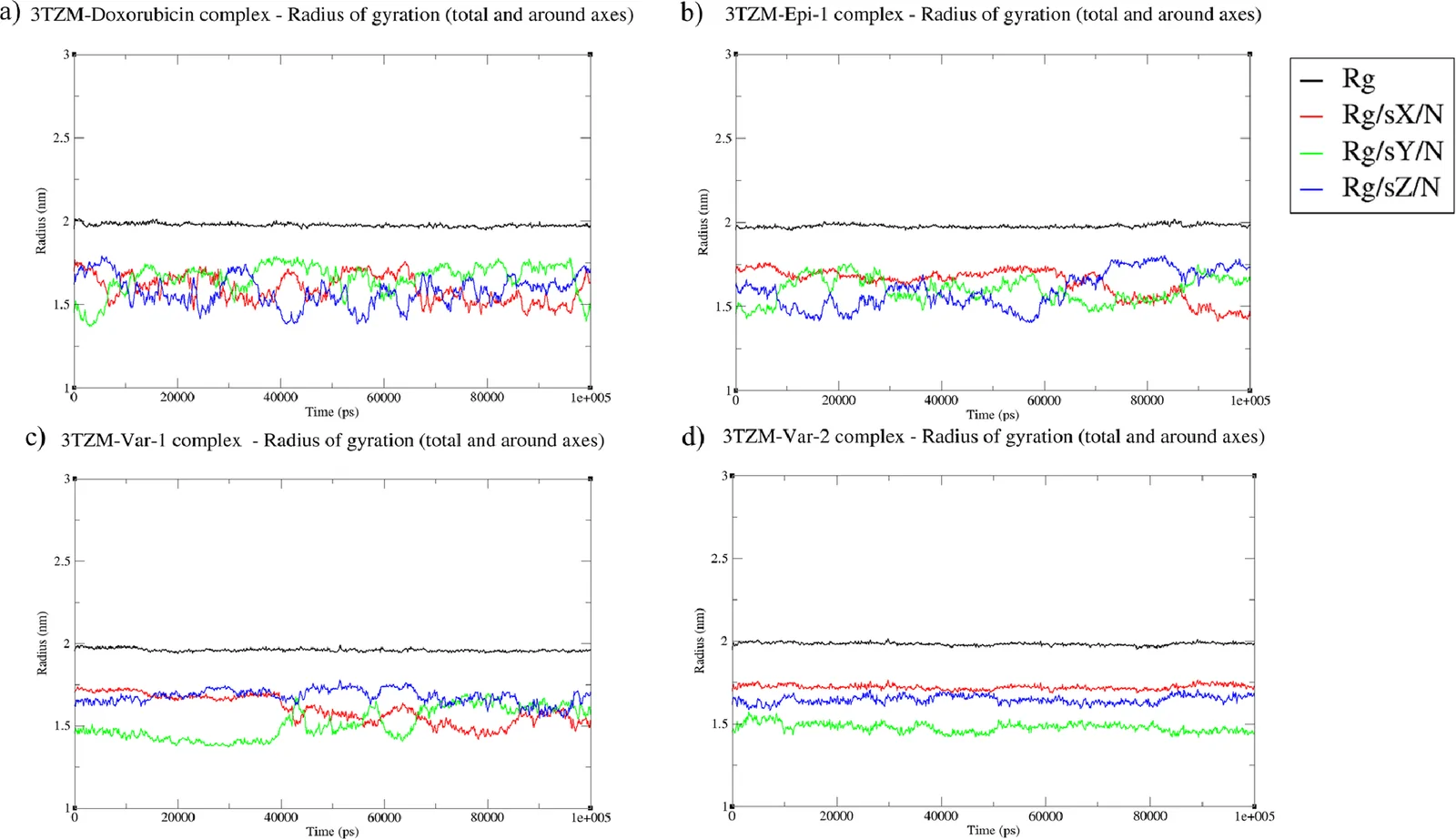
Radius of gyration (Rg) analysis of 3TZM-ligand complexes during 100 ns molecular dynamics simulation. (a-d) Time evolution of the total radius of gyration (Rg) and its components along the principal axes (Rg_x, Rg_y, and Rg_z) for the 3TZM receptor in complex with (**a**) doxorubicin, (**b**) Epi-1, (**c**) Var-1, and (**d**) Var-2. All systems exhibit relatively stable Rg profiles throughout the simulation period, indicating maintenance of structural compactness and preservation of the receptor’s tertiary fold. Minor fluctuations observed along individual axes reflect localized conformational adjustments rather than global unfolding, suggesting that ligand binding does not induce significant structural expansion or destabilization of the protein

Four receptor-ligand systems were simulated for 100 ns: 3TZM-doxorubicin, 3TZM-Epi-1, 3TZM-Var-1, and 3TZM Var-2. Structural stability and conformational behaviour of these complexes were assessed by examining trajectory-derived parameters including backbone deviation, residue flexibility, structural compactness, intermolecular hydrogen bonding, and solvent exposure (Figs. 3 and 4). Average RMSD, RMSF, Rg, and SASA values (mean ± SD) calculated over the equilibrated 20–100 ns window for each of the four systems are summarized in Table S1, providing quantitative support for the stability trends described above. Representative protein–ligand interaction snapshots extracted at the start (0 ns) and end (100 ns) of the production trajectory for each complex are provided in Figure S6, illustrating that the principal binding-pocket contacts identified at the beginning of the simulation are retained at its conclusion.

#### Structural stability of the complexes

The backbone deviation profiles indicate that all complexes underwent a short equilibration phase during the initial portion of the trajectory before stabilizing for the remainder of the simulation. Once equilibrium was reached, the systems fluctuated within a relatively narrow deviation range, suggesting that the receptor maintained a stable structural framework throughout the simulation.

Among the systems analysed, the 3TZM-doxorubicin complex exhibited the most stable trajectory with minimal fluctuations. The peptide-bound complexes also maintained consistent deviation profiles, indicating that the peptides remained accommodated within the receptor cavity during the simulation. Notably, the 3TZM-Var-2 complex displayed slightly lower fluctuations when compared with the Epi-1 and Var-1 complexes, suggesting improved dynamic stability of this Var-2 within the receptor binding region.

#### Residue-level flexibility

Analysis of residue fluctuations revealed that mobility was predominantly localized in terminal segments and loop regions of the receptor, which are characteristically flexible structural elements. In contrast, residues forming the catalytic pocket exhibited comparatively restrained motion throughout the trajectory.

The presence of the peptide ligands did not induce significant destabilization within the active site region. Instead, the binding pocket retained a relatively rigid conformation across all complexes. The Var-2 complex showed slightly reduced fluctuations near the binding interface relative to Epi-1 and Var-1, indicating a more stable interaction environment around the catalytic residues.

#### Structural compactness of the receptor

The overall compactness of the receptor remained largely unchanged throughout the simulation for all complexes. The structural packing of the protein core was preserved over the entire trajectory, with only minor variations that correspond to normal conformational breathing motions of the protein.

Importantly, binding of the peptide ligands did not induce measurable expansion or destabilization of the receptor fold. The Epi-1, Var-1, and Var-2 complexes displayed compactness profiles comparable to that of the doxorubicin-bound system, indicating that peptide association does not perturb the global structural organization of the receptor.

#### Intermolecular hydrogen bonding

The stability of ligand binding was further reflected in the persistence of hydrogen bond interactions within the receptor cavity. Throughout the simulation trajectory, all complexes retained multiple hydrogen bond contacts between ligand functional groups and residues lining the binding pocket.

Among the peptide complexes, Var-2 demonstrated comparatively more persistent hydrogen bonding interactions. These contacts were maintained over extended portions of the trajectory, suggesting stronger stabilization of the peptide within the receptor cavity and consistent engagement with key residues involved in ligand recognition.

#### Solvent accessibility and structural exposure

The solvent exposure profiles of the receptor remained relatively stable during the simulation, indicating that ligand binding did not induce significant rearrangements of the protein surface. Minor variations observed during the trajectory correspond to transient conformational adjustments typical of protein dynamics rather than major structural transitions.

Overall, the solvent exposure patterns were comparable across all complexes, further supporting the observation that ligand association did not disrupt the structural integrity of the receptor.

### Anticancer property of Epi-1, Var-1 and Var-2

Epi-1 and its variants designed by lysine substitution in histidine and alanine to enhance the peptide’s positive charge and amphipathic nature were modified. Var-1 was created by replacing histidine residues with lysine at position 5, 12 and 18. Var-2 was generated by replacing the alanine in the 13th position of Var-1 as shown in previous studies [18]. Substitution of lysine was chosen since lysine repeats enhanced antimicrobial activity [43].

**Fig. 5:**
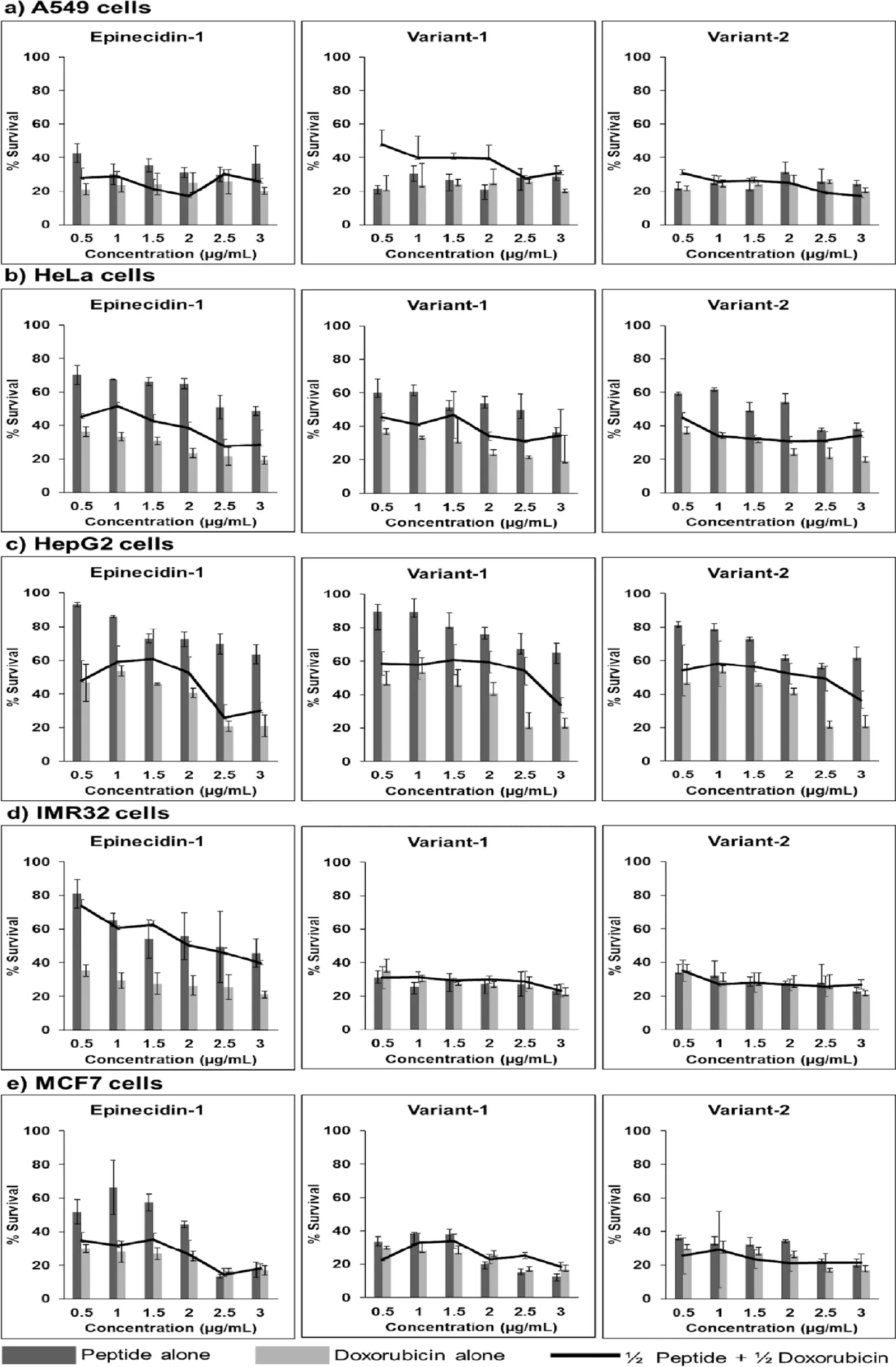
Dose-dependent effects of Epi-1 and its variants treatment (24 h) on survival (MTT assay) across multiple cell lines (**a**) A549 lung cancer cells, (**b**) HeLa cervical cancer cells, (**c**) HepG2 liver cancer cells, (**d**) IMR-32 neuroblastoma cells and (**e**) MCF-7 breast cancer cells. Bar graphs show percentage survival of cells treated with increasing concentrations (0, 0.5, 1, 1.5, 2, 2.5, and 3 µg/mL) of Epi-1, Var-1, and Var-2. The growth of cells without any treatment is used for control with 100% survival. Each row represents a distinct experimental condition against the cell line tested and each column corresponds to one of the three peptides. Data are presented as mean ± standard deviation from replicate experiments. The dark grey bars represent the peptide alone, the light grey bar represent the doxorubicin alone and the black line graph represent the half the concentration of peptide and doxorubicin alone. For example, 0.5 µg/mL panel represent 0.25 µg/mL peptide and 0.25 µg/mL of Doxorubicin

The anticancer property of the peptides alone and in combination with doxorubicin were determined by MTT assay against cancer cells such as HeLa, MCF-7, IMR-32, HepG2 and A549 cells [44, 45]. Cell death was quantified and compared across conditions shown in Fig. 5. For peptide alone treatments, all three peptides exhibited dose-dependent cytotoxicity across the tested cell lines. Epi-1 reduced survival significantly at concentrations ≥ 2 µg/mL in most cell types, while Var-1 showed a more gradual decline in viability, maintaining higher death rates at 2 to 3 µg/mL concentrations. Var-2 demonstrated enhanced potency, with significant reductions in survival observed at concentrations as low as 1 µg/mL in selected cancer cell lines. Doxorubicin treatment resulted in substantial cytotoxicity in cancer cell lines, with survival dropping below 30% at 1 µg/mL.

Combination treatment with peptides and doxorubicin revealed additive and synergistic effects. In cancer cell lines, the combination of Var-2 and doxorubicin led to significantly enhanced cytotoxicity compared to either of them alone, with survival rates falling below 10% at combined concentrations of 1 µg/mL each. Epi-1 combined with doxorubicin also showed improved efficacy. Notably, Var-1 combined with doxorubicin maintained moderate cytotoxicity in cancer cells while preserving higher viability in non-cancerous lines, suggesting potential for selective therapeutic application [35].

Epi-1 showed a synergistic effect with doxorubicin in A549, HeLa, HepG2, and MCF-7 cells, while an additive effect was observed in IMR-32 cells. Var-1 exhibited synergism with doxorubicin in A549 and MCF-7 cells, but showed weaker interaction in HeLa and HepG2 cells. Var-2 displayed synergistic effects with doxorubicin in A549, HeLa, HepG2, and MCF-7 cells, with the strongest additive effect observed in MCF-7 cells. Importantly, no antagonistic interactions were detected in any combination. The peptide-doxorubicin combinations achieved near-complete growth inhibition at a concentration of 3 µg/mL across all cell lines, with enhanced cell death suggesting distinct mechanisms of action. Furthermore, the maximal inhibitory effect of doxorubicin was achieved at half the usual concentration when used in combination, supporting the hypothesis that peptides enhance drug accumulation and permeability in cancer cells [46], potentially bypassing drug efflux mechanisms. While these findings suggest promising synergism, especially with Epi-1 and Var-2, further studies are needed to confirm the underlying mechanisms [47]. Overall, the peptide-doxorubicin combinations significantly reduced cancer cell growth, with the effect being twice as potent in HeLa and HepG2 cells and requiring only half the dose of doxorubicin in A549 and MCF-7 cells. The combination effect in IMR-32 cells was less pronounced.

### Quantitation analysis of AMP and doxorubicin combinations

Across the tested cancer cell lines, combination responses showed distinct patterns of Bliss‑independence interaction. HeLa and MCF-7 demonstrated the strongest positive inhibition showing robust synergistic interactions across the peptides. Epi-1 and Var-1 combination produced the highest synergy scores with doxorubicin.

A549 exhibited selective synergy with Var-1 and doxorubicin combination, Epi-1 and Var-2 combination with doxorubicin only modest or borderline‑synergistic effects. IMR‑32 showed strong synergy for Epi-1 and Var-1 with doxorubicin. Var‑2 combination showed additive effect (Table 4).

**Fig. 6:**
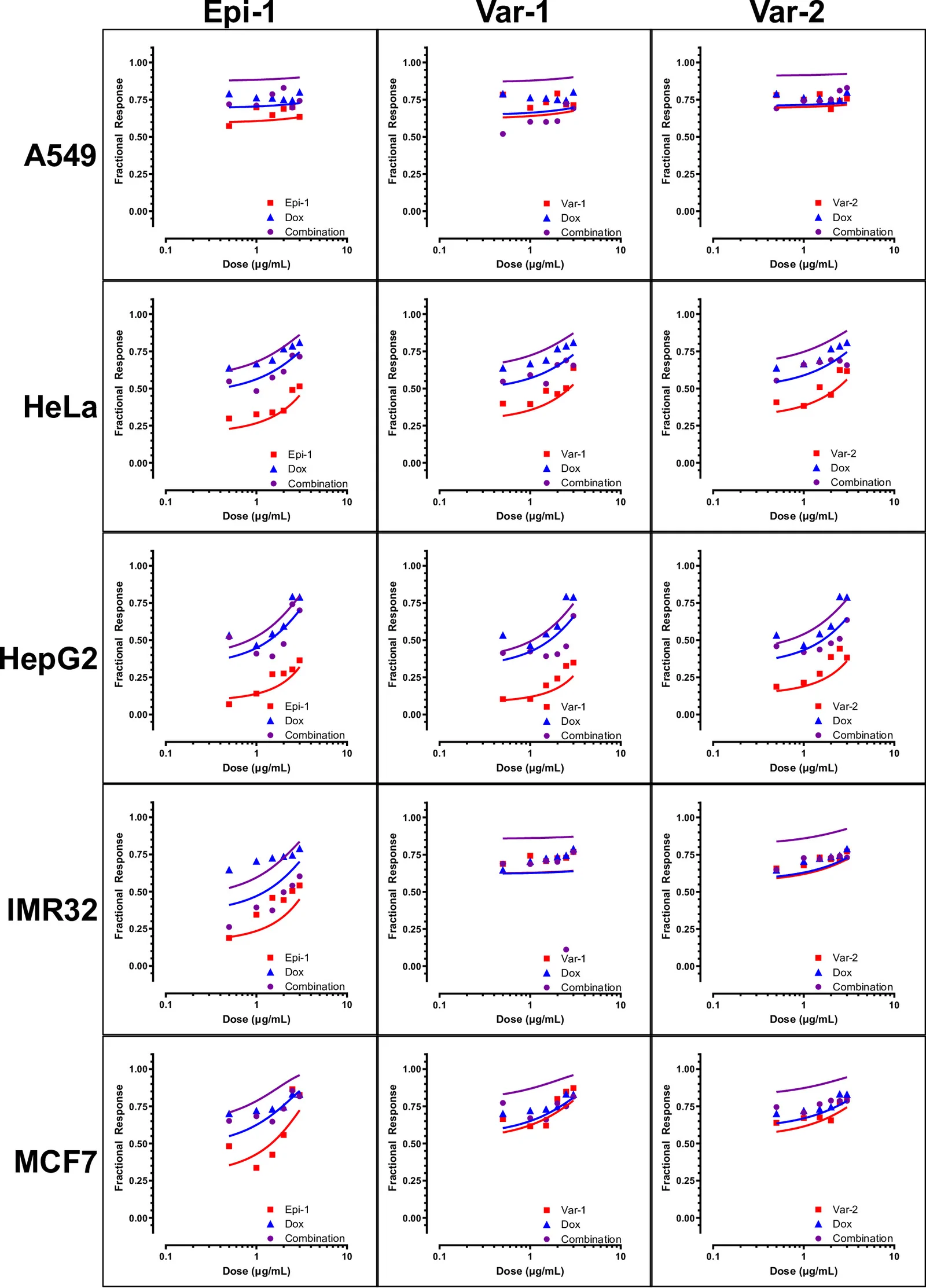
Bliss Independence interaction analysis of Epi‑1, Var‑1, and Var‑2 with doxorubicin across the cancer cell lines. Each panel illustrates the response profile of individual peptide with doxorubicin

In contrast, HepG2 displayed the lowest synergy potential, with most combinations falling within the additive range and antagonism for Var‑2 with doxorubicin (Fig. 6). Interactions were classified by comparing the observed combined effect (Eobs) with the Bliss-predicted effect (EBliss): synergistic when Eobs > EBliss, additive when Eobs = EBliss, and antagonistic when Eobs < EBliss.

**Table 4:** Comparative logEC₅₀ values for Epi‑1, Var‑1, Var‑2, and Doxorubicin of the cancer cell lines

	logEC50 values			
	Epinecidin-1	Variant-1	Variant-2	Doxorubicin
A549	−6.822	−6.477	−22.61	−24.522
HeLa	3.45	2.69	2.32	0.2
HepG2	4.4	5.2	4.3	1.61
IMR-32	3.4	−18.36	−1.151	−1.34
MCF-7	1.466	−0.2292	−0.5646	−1.357

### Differential AO/EtBr staining of A549, HeLa and IMR 32 cells after treatment with Epi- 1, Var-1, Var-2 and doxorubicin

Peptide-induced cell death was assessed using differential DNA staining. To evaluate whether Epi-1 and its variants induce apoptosis, nuclear changes associated with AO/EtBr fluorescence staining were compared with control and peptide treated cells on A549, HeLa, HepG2 and IMR 32 cell lines. Cells treated with 3 µg/mL Doxorubicin served as the positive control.

**Fig. 7:**
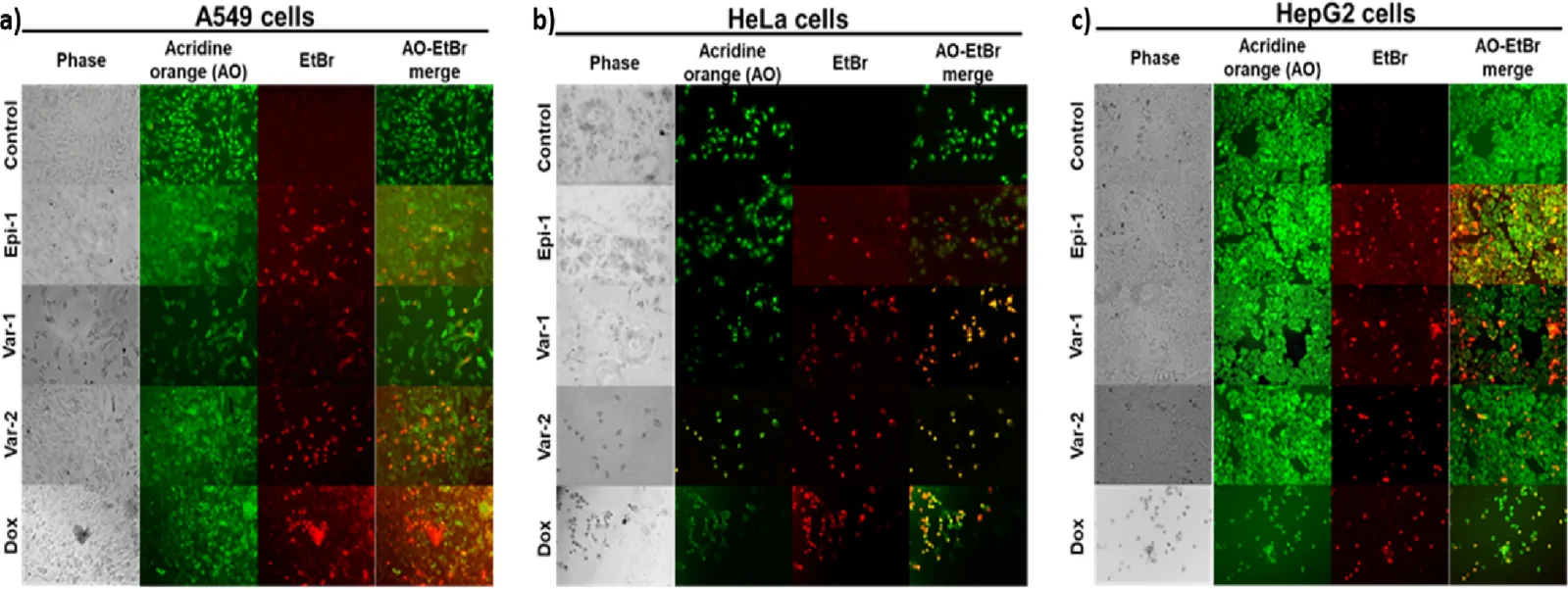
Morphologic changes of (**a**) A549, (**b**) HeLa, and (**c**) HepG2 cells with acridine orange/ethidium bromide (AO/EtBr) staining. Cells treated with DMEM without any peptide treatment served as control. Epi-1, Var-1, Var-2 and doxorubicin was added to cells at 3 µg/mL and incubated for 24 h. AO/EtBr staining produced green fluorescence in live cells and orange to red nuclei in apoptotic cells

In all tested cancer cell lines Fig. 7. (top panel left to right), untreated control cells exhibited healthy morphology characterized by uniform shape, intact membranes, and dense distribution. Acridine Orange (AO) staining of control cells revealed strong green fluorescence, indicating high cell viability [48]. Ethidium Bromide (EtBr) staining showed minimal red fluorescence, suggesting negligible cell death. The combined AO/EtBr staining image shows predominantly green fluorescence, confirming a largely viable and healthy cell population [49]. Phase contrast and AO/EtBr fluorescence images were used to assess morphological changes and cell viability in peptide treated cells.

For A549 cells, the phase contrast image of Epi-1 shows peptide induced mild morphological changes, suggesting early cellular stress. The AO staining shows slightly reduced green fluorescence, suggesting moderate viability. The EtBr staining shows more red staining indicating cytotoxicity. The merging of AO and EtBr images shows orange and red fluorescence indicating apoptosis and cell death [50]. For Var-1, the number of cells in the field is very low compared to control and Epi-1 or Var-2. There are more red fluorescence indicating cytotoxicity. The merge of AO and EtBr images shows prominent red color suggesting cytotoxicity. For Var-2, many cells appear rounded or fragmented, similar to apoptotic bodies. A very strong red signal, indicating extensive cell death. The merged image shows dominant bright yellow/orange regions, suggesting a cytotoxic effect. Doxorubicin-treated cells exhibit severe morphological disruption, including cell lysis, detachment, and debris [51]. Green fluorescence is minimal, indicating low viability, while the merged image shows intense yellow/orange fluorescence, confirming a broad range of apoptotic cell death [52].

For HeLa cells, the control cells without peptide treatment appeared green (Fig. 7) while the epi-1 treated cells were observed red indicating the cell death by cytotoxicity. Hela cells treated with variants and doxorubicin show decreased cell numbers compared to control cells. For the variant treated cells, Acridine orange shows faint green fluorescence and EtBr shows bright red fluorescence. The merge of AO and EtBr show yellow and orange fluorescence indicating apoptotic mode of cell death. For Doxorubicin-treated cells, the merge of AO and EtBr shows a mixed population of red, green and yellow-orange cells [53].

HepG2 control cells are mostly viable, showing strong green fluorescence. The AO panels of peptide treated cells did not show reduction in number of cells [54]. This result also correlates with the MTT assay as shown in Fig. 7. Though there is no reduction in the number of cells after peptide treatment, the EtBr and AO/EtBr merged panels show strong red color [55]. This shows molecular events of cell death; however, the dead cells did not detach from the surface of the culture plate. Epi-1 and Var-1 showed high numbers of red cells in the merged field. Var-2 had a smaller number of red fluorescent cells compared to Epi-1 and Var-1 in the merged field; However, it contained few yellow orange fluorescence cells in addition to the red fluorescent cells. The doxorubicin treated cells show decreased cell number compared to control and peptide treatment. A significant number of red fluorescent cells are visible in the EtBr panel. In the merged panel, these red cells appear yellow fluorescence cells due to the overlap of green and red fluorescence, indicating apoptotic cell death.

### ROS detection in Hela cells after peptides treatment

**Fig. 8:**
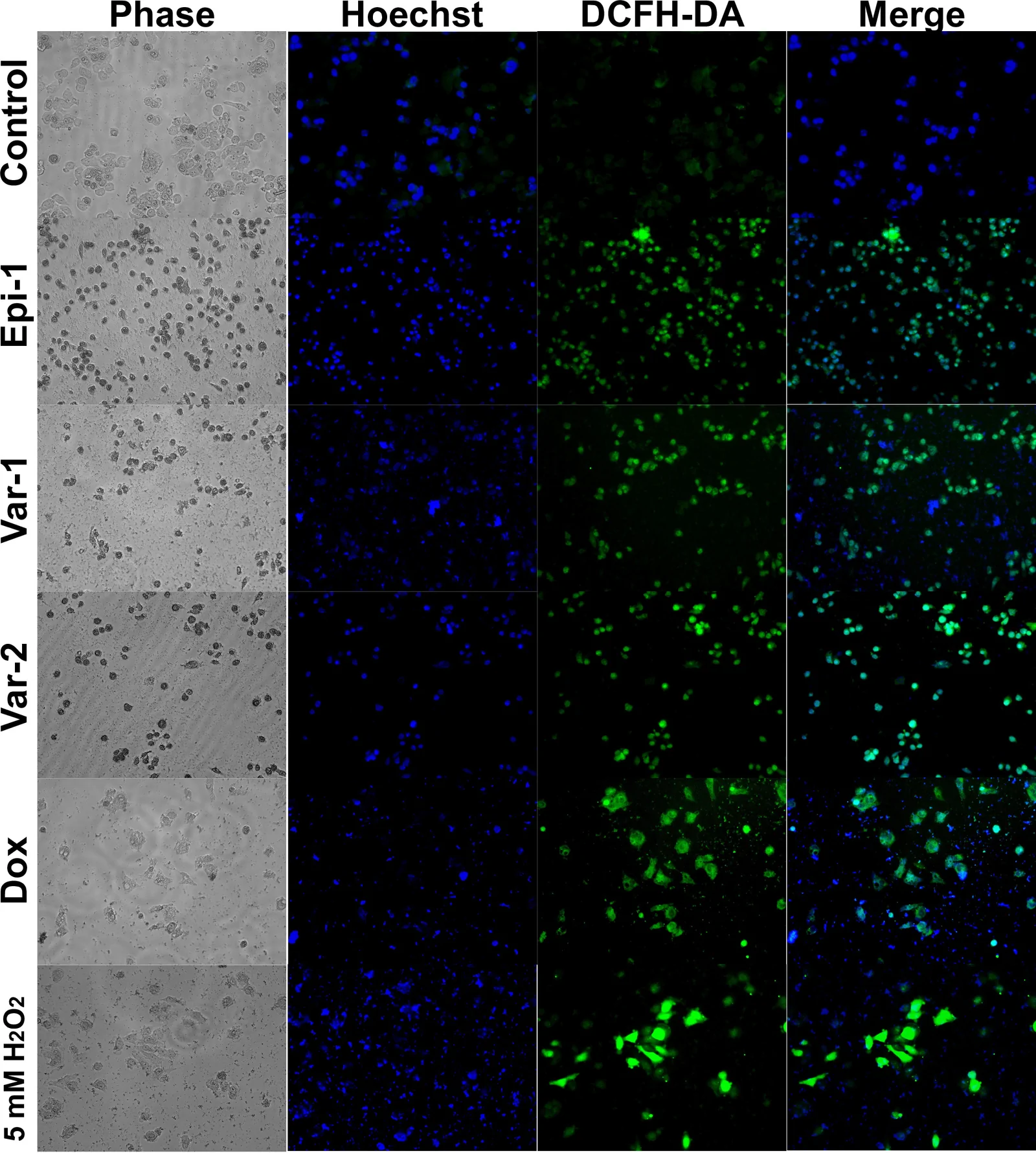
Epi-1 and its variants induced production of reactive oxygen species (ROS) in HeLa cells treated with 3 µg/mL for 6 h. Intracellular ROS were detected by staining with dichlorofluorescein diacetate (DCFH-DA) that emits fluorescence upon oxidation. Representative fluorescent microscopic image profile of the peptide treated, and control cells are illustrated along with 5 mM H₂O₂ as positive control

The microscopy image presents a comparative analysis of cellular responses to DCFH-DA after treating with peptides. The top row represents control cells without any treatment. Doxorubicin, and 5 mM H₂O₂, were used as controls to induce reaction oxygen species (ROS). Phase contrast and Hoechst nuclear staining panels show the presence and morphological characteristics of the cells. Control cells exhibit normal morphology and minimal ROS. Epi-1 induces mild oxidative stress with moderate ROS levels [41]. Var-1 and Var-2 show progressively stronger cytotoxic effects, with increased ROS and altered cell morphology. Doxorubicin-treated cells display disrupted structure and high ROS, indicating apoptosis (Fig. 8). The 5 mM H₂O₂ condition results in severe cell damage and maximal ROS, acting as a positive control for oxidative stress [42].

### Toxicity assay of peptides against non-cancerous HEK cells

**Fig. 9:**
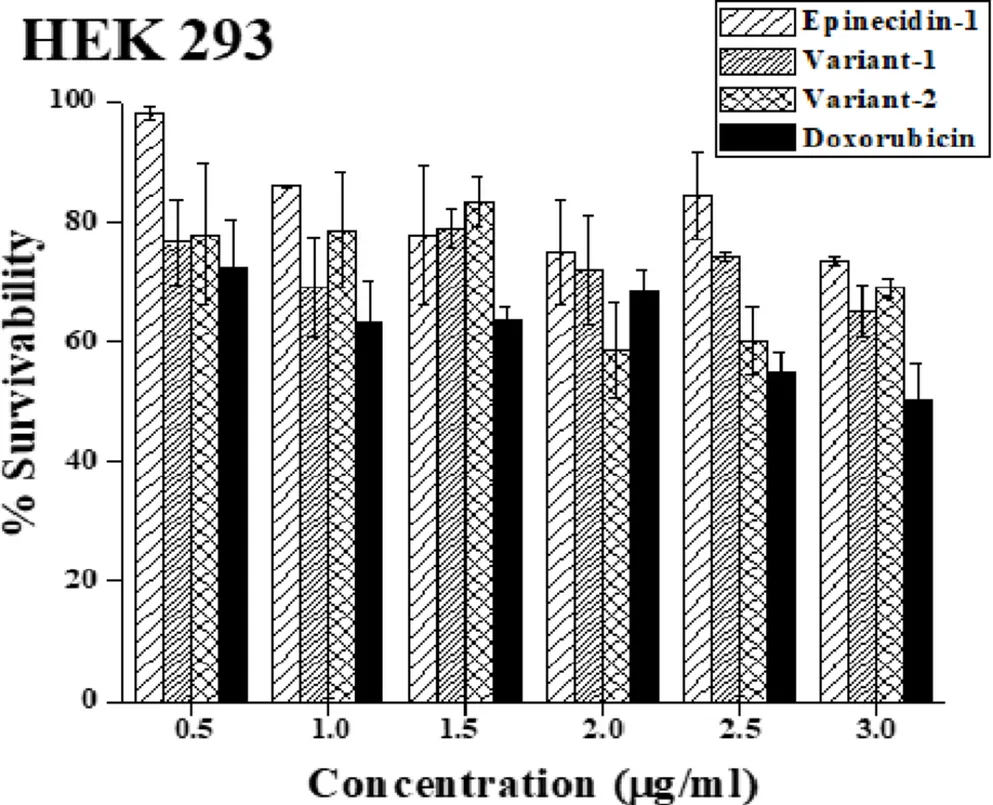
Effects of Epi-1, its variants, with Doxorubicin on HEK cell viability. Bar graph depicting treatment with Epi-1 (diagonal lines), Var-1 (horizontal lines), Var-2 (crosshatch), and Doxorubicin (solid black). Cell survivability was assessed by MTT assay post-treatment and expressed as percentage relative to untreated controls. Doxorubicin consistently induced the greatest reduction in viability across all concentrations. Epi-1 and its variants showed variable effects, with Var-2 exhibiting intermediate cytotoxicity. Data represent mean ± SD from three replicates

To assess the cytotoxicity of the peptides on non-cancerous cells, HEK cells were treated with Epi-1, Var-1, and Var-2 at the same concentrations used for the cancer cell lines. Figure 9 presents the percentage of HEK cell survivability following treatment, with Doxorubicin included as a control. Epi-1 exhibited the highest cell viability among the tested peptides, indicating lower cytotoxicity toward non-cancerous cells. In comparison, Var-1 and Var-2 showed reduced survivability, with Var-1 demonstrating greater cytotoxicity than Var-2.

## Discussion

The molecular docking results offer a mechanistic framework for understanding the predicted anticancer potential of Epi-1 and its lysine-modified variants. The in silico docking predictions demonstrated a clear qualitative concordance with the observed in vitro potencies. Var-2, which exhibited the most favorable binding free energies across the oncogenic receptor panel including MerTK (7OLX), EphA3 (2QO9), TGF-β receptor I/ALK5 (3TZM), TrkA/NTRK1 (4AOJ), and the progesterone receptor (1A28) consistently produced superior cytotoxic responses and stronger synergism with doxorubicin in MTT assays across HeLa, MCF-7, and A549 cells, with comparatively moderate effects in IMR-32 and HepG2 cells, mirroring its docking profile. Although peptide-mediated cytotoxicity is multifactorial and influenced by membrane disruption and intracellular ROS generation, the concordance between enhanced receptor binding affinity and experimental potency supports the predictive value of the docking analysis guided by lysine substitution. Strategic lysine substitution enhances electrostatic complementarity and stabilizes receptor interactions without disrupting the native α-helical conformation. Var-1 exhibited intermediate affinities, indicating that charge distribution and amphipathic balance play a decisive role in receptor recognition and complex stability. It should be noted that these receptor-mediated mechanisms are inferred from docking and molecular dynamics correlations alone, and remain hypothetical until tested directly in cells. Validation by receptor knockdown or competition assays, surface plasmon resonance, or fluorescence co-localization studies will be necessary to confirm receptor engagement by the peptides.

Although doxorubicin retained the strongest overall binding, the comparable affinities of Var-2 highlight its potential as an active molecular scaffold capable of engaging multiple oncogenic targets. The docking data imply that these peptides can associate with receptor regions implicated in growth signaling and cell-survival regulation, potentially modulating receptor activity or hindering ligand access. Such interactions could contribute reduced proliferative signaling and increased apoptotic sensitivity in tumor cells.

These in-silico findings provide the structural rationale for the in vitro observations described in the subsequent sections. The enhanced receptor affinities of Var-2 correlate with its superior cytotoxic and synergistic responses when combined with doxorubicin, supporting the notion that receptor-level engagement facilitates drug uptake and amplifies intracellular cytotoxicity. Moreover, the amphipathic nature of the peptides likely promotes transient membrane perturbation, further enhancing drug internalization and apoptotic signaling as seen with cell penetrating peptides [56], such as dNP2 [57] L-K6 [58], MTS-R_8_H_3_ [59].

Together, the docking predictions and experimental outcomes reinforce the hypothesis that rational amino-acid substitution in Epi-1 can fine-tune both receptor binding and membrane activity *via* enhancing charge, providing a dual mechanism of action that improves therapeutic efficacy in cancer models.

To further examine the stability of the selected receptor-ligand complexes under dynamic conditions, molecular dynamics simulations were performed for the 3TZM system, which exhibited the most favourable binding energies and consistent interaction profiles during docking. Given its established role in TGF-β-mediated oncogenic signaling, this receptor was considered suitable for detailed dynamic evaluation [60]. Consistent with trajectory analyses, all complexes attained equilibrium during the initial phase and remained stable over the 100 ns simulation, indicating preservation of the receptor structure upon ligand binding. The doxorubicin-bound complex displayed minimal deviation, while the peptide-bound systems maintained comparable stability, with Var-2 exhibiting relatively lower fluctuations within the binding region.

Analysis of RMSF, Rg, SASA and intermolecular hydrogen bonding collectively indicated that structural flexibility was largely confined to loop and terminal regions, whereas residues within the catalytic pocket remained structurally constrained [61, 62]. The Rg and solvent accessibility profiles remained stable throughout the simulation, confirming maintenance of structural compactness without significant conformational rearrangements. Persistent hydrogen bonding further supported stable ligand retention within the binding pocket, with comparatively greater interaction stability observed for Var-2. Together, these observations indicate that the peptide-receptor complexes remain structurally stable under dynamic conditions, consistent with the docking predictions.

Across five cancer lines, peptide–doxorubicin combinations lowered cell viability more than monotherapies at matched total concentrations, with Var- 2 being the most consistently effective. In contrast, HEK 293 cells showed comparatively higher viability with peptides than doxorubicin. Given the previously reported antimicrobial properties of Epi-1 and its variants, we investigated their potential anticancer activity through in vitro assays using various cancer cell lines [36, 46]. Cancer cells share several biophysical and biochemical features with microbial cells, including a net negative charge due to phosphatidylserine overexpression [13], and the presence of sialic acid, hyaluronan, and related glycosaminoglycans in their membranes and microenvironment [10]. These characteristics contribute to the heightened susceptibility of malignant cells to cationic antimicrobial peptides (AMPs).

To explore this, we tested Epi-1 and its lysine-substituted variants for anticancer efficacy. Previous studies on gaegurin-derived AMPs demonstrated that lysine substitution enhanced antimicrobial and anticancer activity. In the variants, lysine substitution led to increased structural rigidity, amphipathicity, and improved secondary structural conformation, which collectively contributed to enhanced its anticancer potency compared to the parent peptide [63].

Both cationic and hydrophobic residues are known to facilitate peptide permeation across mitochondrial membranes [36, 46]. To investigate the mechanism of action, we concurrently assessed reactive oxygen species (ROS) generation and acridine orange/ethidium bromide (AO/EtBr) staining. As intracellular DCFH-DA is oxidized by highly reactive species, including peroxynitrite (ONOO⁻) and peroxidase-mediated derivatives of hydrogen peroxide, the increase in green fluorescence observed following antimicrobial peptide treatment reflects an elevation in intracellular oxidative stress, consistent with activation of downstream apoptotic signaling pathways [3, 64, 65].

AO/EtBr staining showed that HeLa cells treated with Epi‑1 and its variants displayed red fluorescence and marked morphological alterations consistent with apoptosis, in agreement with previous reports demonstrating Epi-1 induces apoptotic cell death [66]. In contrast, HepG2 cells showed minimal response to peptide treatment, while doxorubicin-treated controls displayed significant cell death. A549 cells demonstrated orange to reddish staining, characteristic of late apoptotic stages. A plausible explanation for this relates to the mode of action of AMPs, which depends largely on charge-based interaction with the plasma membrane. Hepatocyte-derived cells maintain a relatively stable resting membrane potential, with little voltage fluctuation compared to other cancer cell types. This stability could limit peptide–membrane engagement and may account for the weaker antiproliferative and synergistic effects of the peptides in HepG2 cell [67]. These observations suggest a pleiotropic mechanism of action, wherein the peptides may interact with extracellular components in a cell-type-dependent manner, potentially bypassing conventional drug resistance pathways.

Importantly, non-cancerous HEK-293 cells were minimally affected by peptide treatment, indicating preferential cytotoxicity toward malignant cells. Future mechanistic imaging studies employing fluorescent-tagged Epi-1 variants may elucidate subcellular localization and real-time membrane interaction dynamics [68, 69].

## Conclusions

The present study concludes Epi-1 and its lysine-modified variants exert measurable anticancer activity in a range of human cancer cell lines. Introducing lysine in place of histidine and alanine increased the net positive charge and amphipathic properties of the peptides, which in turn improved their interaction with cancer-cell membranes and associated receptor sites. When combined with doxorubicin, these peptides produced clear synergistic responses in HeLa, MCF-7, and A549 cells, whereas IMR-32 and HepG2 cells exhibited an additive effect. Notably, none of the combinations resulted in antagonism.

Docking studies complemented the experimental findings, with Var-2 exhibiting the most stable interactions across the corresponding oncogenic receptors, mirroring its enhanced cytotoxicity and synergistic behaviour. These combined observations suggest that lysine substitution strengthens both receptor-level engagement and membrane-disruptive activity, thereby mediating cell death. Taken together, the data identify the variants as the most effective derivatives, with a favourable balance of charge, helicity, and amphipathicity that supports its application in combination-based cancer therapy. Molecular dynamics simulations further confirmed the stability of these interactions under dynamic conditions, with Var-2 maintaining consistent conformational stability and interaction persistence within the receptor binding site. Future work will focus on receptor-engagement validation studies, in vivo efficacy and toxicity evaluation, and formulation optimization (e.g., nanocarrier-based delivery) of Var-2 as the lead candidate, as the next logical steps toward its translational development.

## Supplementary Information

Below is the link to the electronic supplementary material.

Supplementary Material 1

Supplementary Material 2

## Data Availability

All data supporting the findings of this study are available within the paper and its Supplementary Information.
