# Supplementary material for: Anticancer effects of alpha-helical peptide epinecidin-1 and its variants in combination with doxorubicin
Source: Med Oncol. 2026 Jul 22;43(9):231. doi: 10.1007/s12032-026-03344-0 (PMC13391716; doi:10.1007/s12032-026-03344-0)
Supplement: Supplementary file 2 — Supplementary Material 2 [file 12032_2026_3344_MOESM2_ESM.docx]

**Anticancer effects of alpha-helical peptide Epinecidin-1 and its variants in combination with doxorubicin**

Sivakumar Jeyarajan^#^ ^1,2^, Sukumar Ranjith^#^ ^1^, Atchyasri Anbarasu ^3^, Indira Kandasamy ^1^, Prahalathan Chidambaram^4^ and Anbarasu Kumarasamy ^1^*


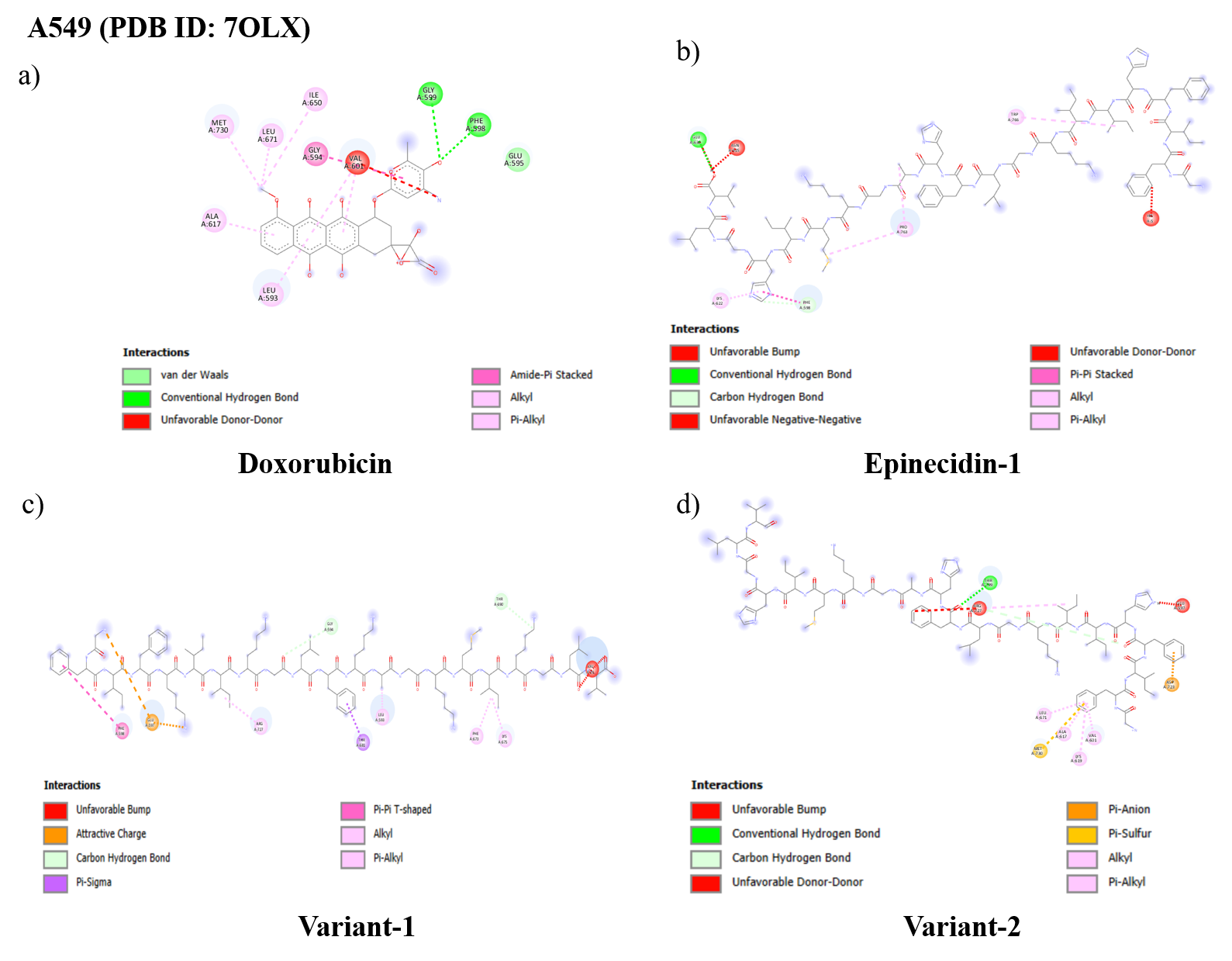
Supplementary Figures

**S1**

**
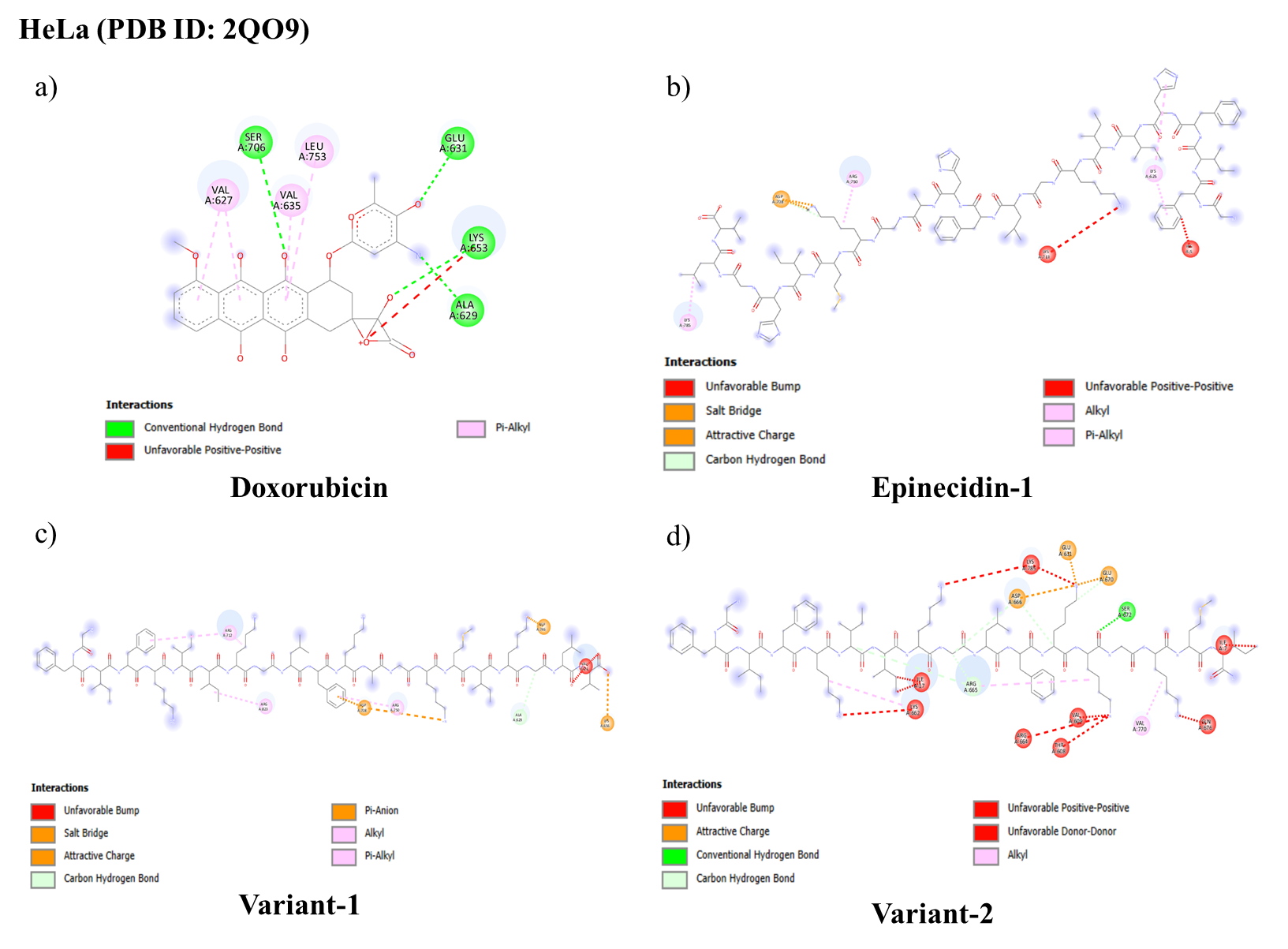
S2**

**
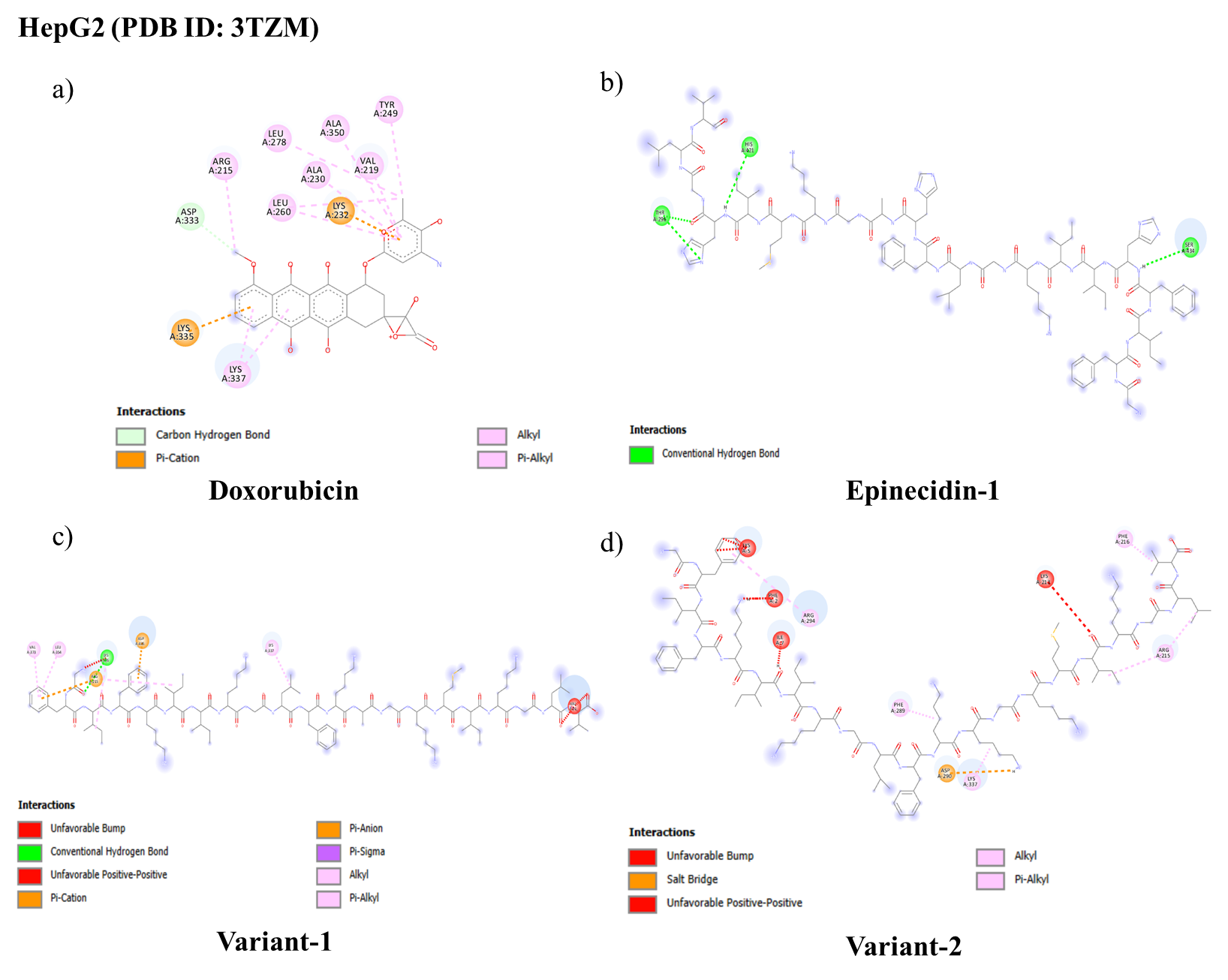
S3**

**
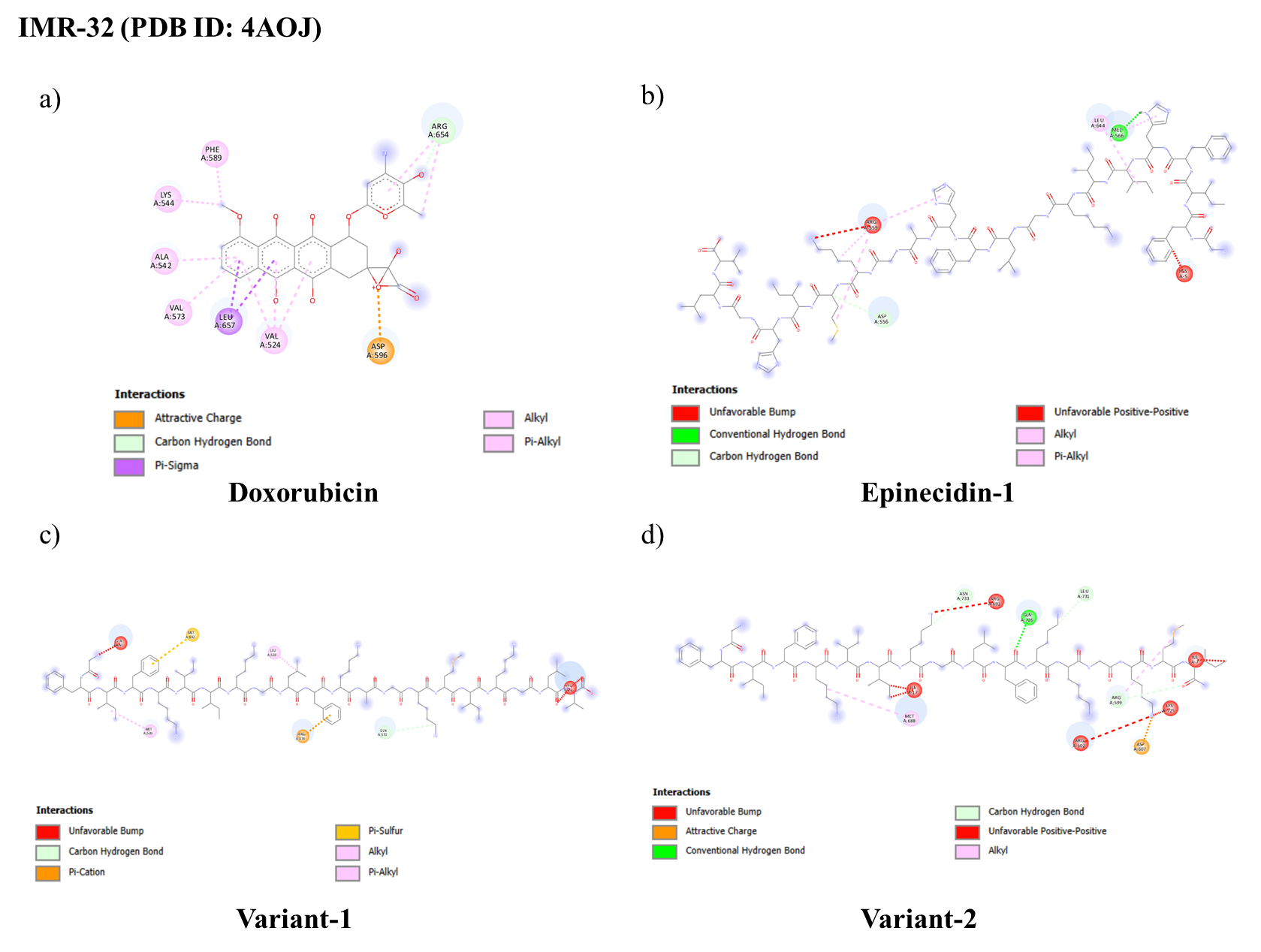
S4**

**
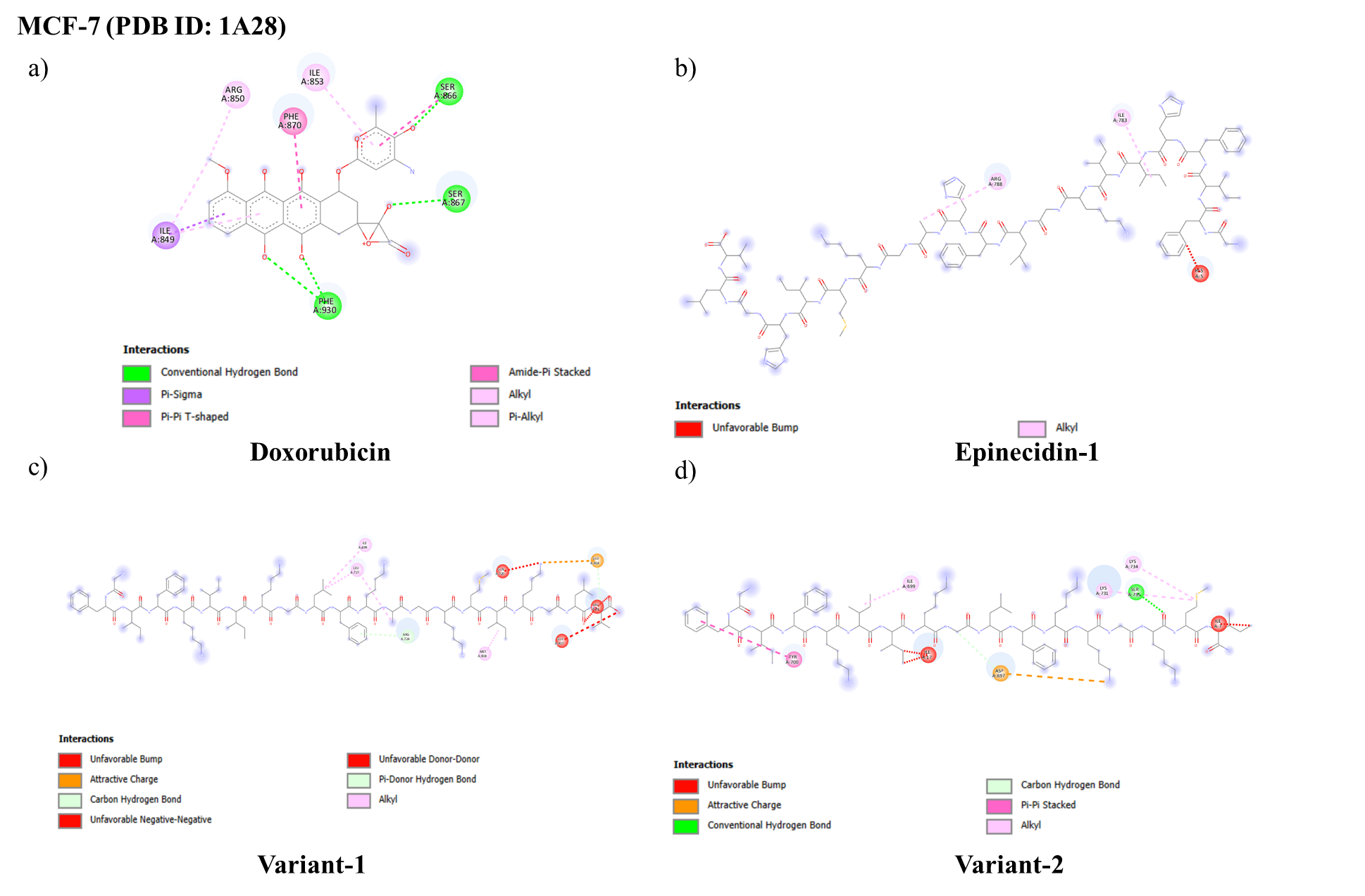
S5**

**S6**


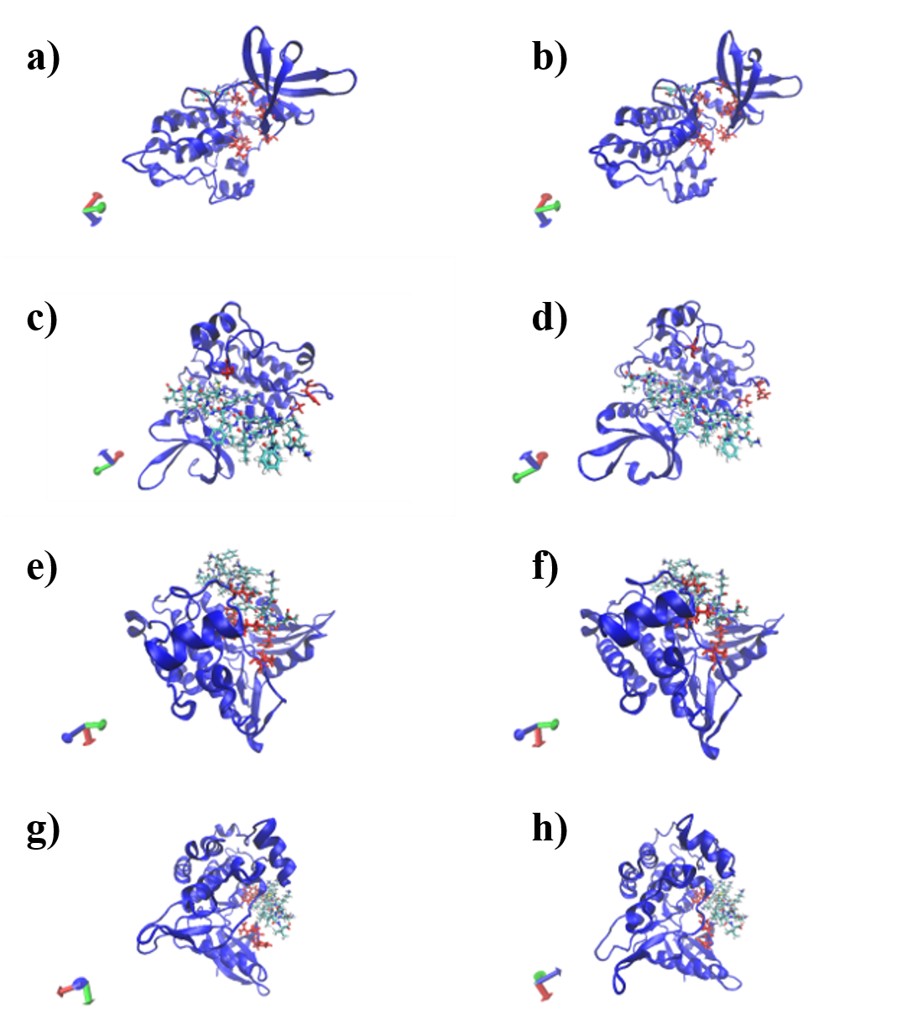


**Figure S1.**

Two-dimensional interaction profiles of ligands docked with the A549-associated receptor (PDB ID: 7OLX) are compared for doxorubicin, Epi-1, and variants (Var-1 & Var-2). Doxorubicin (a) engages predominantly through van der Waals contacts and conventional hydrogen bonds, with a notable donor–donor conflict. Epi-1 (b) forms multiple hydrogen bonds and hydrophobic interactions, although several steric clashes and donor-donor contacts are evident. In contrast, Var-1 and Var-2 (c, d) display expanded interaction networks, comprising carbon hydrogen bonds, alkyl and π-alkyl contacts, π-π stacking, and electrostatic contributions such as π-anion and π-sulfur interactions. The interaction patterns point to improved binding complementarity in the variants relative to both doxorubicin and the parent peptide.

**Figure S2.**

Ligand interaction maps for the EphA3 receptor kinase domain from HeLa – associated receptor (PDB ID: 2QO9) reveal clear differences in binding behaviour among the tested ligands. Doxorubicin (a) is stabilized mainly through hydrophobic contacts, van der Waals interactions, and conventional hydrogen bonds. Epi-1 (b) forms salt bridges and carbon hydrogen bonds, but also shows unfavorable positive-positive interactions within the binding interface. In contrast, Var-1 and Var-2 (c, d) display a broader and more integrated interaction profile, including π-alkyl, π-π stacking, π-sulfur, π-cation, and halogen contacts, supported by consistent hydrogen bonding. The expanded interaction landscape in the variants suggests improved binding complementarity and specificity towards the EphA3 receptor.

**Figure S3.**

Docking interaction profiles with the HepG2-associated receptor (PDB ID: 3TZM) highlight progressive changes in binding architecture. Doxorubicin (a) forms carbon hydrogen bonds, π-alkyl interactions, and π-π stacking within the binding pocket. Epi-1 (b) engages through conventional hydrogen bonds and hydrophobic contacts. Var-1 and Var-2 (c, d) exhibit more intricate interaction networks, incorporating π-donor hydrogen bonds, π-anion interactions, salt bridges, and π–sigma contacts, alongside occasional steric constraints. These features suggest improved ligand–receptor complementarity in the variants.

**Figure S4.**

Interaction maps for the IMR-32-associated receptor (PDB ID: 4AOJ) further distinguish the binding behaviour of the variants. Doxorubicin (a) is characterised by π-anion, π-alkyl, and π-π T-shaped interactions. Epi-1 (b) forms carbon hydrogen bonds but shows unfavorable positive-positive contacts. Var-1 and Var-2 (c, d) establish more extensive interaction networks, including π-π stacking, π-cation, π-sulfur, alkyl, and halogen contacts, supported by hydrogen bonding. The overall interaction profile suggests increased stability of variant-receptor complexes.

**Figure S5.**

Docking interactions with the MCF-7-associated receptor (PDB ID: 1A8) show clear differences in binding organisation. Doxorubicin (a) forms π–hydrogen bonds alongside hydrophobic contacts. Epi-1 (b) is limited to carbon hydrogen bonding and exhibits steric clashes. Var-1 and Var-2 (c, d) display more cohesive interaction networks, including π-π stacking, π-alkyl and alkyl contacts, amide–π stacking, and conventional hydrogen bonds, with fewer unfavorable interactions. These features support improved binding affinity and specificity of the engineered variants in the breast cancer model.

**Figure S6.**

Representative protein–ligand interaction snapshots of the four 3TZM complexes extracted from the beginning (0 ns) and end (100 ns) of the 100 ns production molecular dynamics trajectory. (a, b) 3TZM–doxorubicin; (c, d) 3TZM–Epinecidin-1 (Epi-1); (e, f) 3TZM–Var-1; (g, h) 3TZM–Var-2. Left-hand panels (a, c, e, g) correspond to the initial (0 ns) conformation, and right-hand panels (b, d, f, h) correspond to the final (100 ns) conformation of each system, rendered from an identical viewing angle within each pair to allow direct visual comparison. The receptor is shown as a blue cartoon, with key contact residues highlighted in red; the ligand (doxorubicin or peptide) is shown in licorice representation. Across all four systems, the ligand remains associated with the same general binding region throughout the simulation, and the principal contact residues identified at 0 ns are still engaged at 100 ns, consistent with stable binding over the simulated timescale. For the Epi-1 complex (c, d), the peptide occupies a broader, more dispersed surface area at 0 ns, consolidating into a more compact binding arrangement by 100 ns, while the Var-1 and Var-2 complexes (e–h) maintain a comparatively localized binding footprint throughout.

**Table S1.**

Average root-mean-square deviation (RMSD), root-mean-square fluctuation (RMSF), radius of gyration (Rg), and solvent-accessible surface area (SASA) for the 3TZM complexes with doxorubicin, Epi-1, Var-1, and Var-2 over the equilibrated portion (20–100 ns) of the 100 ns molecular dynamics trajectory.

| **System** | **RMSD (nm)** | **RMSF (nm)** | **Rg (nm)** | **SASA (nm²)** |
| --- | --- | --- | --- | --- |
| 3TZM–Doxorubicin | 0.149 ± 0.016 | 0.081 ± 0.042 | 1.97 ± 0.01 | 154.0 ± 2.2 |
| 3TZM–Epi-1 | 0.186 ± 0.014 | 0.085 ± 0.051 | 1.98 ± 0.01 | 176.0 ± 2.9 |
| 3TZM–Var-1 | 0.127 ± 0.012 | 0.072 ± 0.037 | 1.96 ± 0.01 | 168.0 ± 3.2 |
| 3TZM–Var-2 | 0.216 ± 0.011 | 0.085 ± 0.048 | 1.98 ± 0.01 | 164.0 ± 2.9 |

*Values are reported as mean ± standard deviation calculated over the equilibrated 20–100 ns window of the 100 ns production trajectory using gmx analyze. Rg - radius of gyration; SASA - solvent-accessible surface area.*
